# Supplementary material for: Tunable afterglow for mechanical self-monitoring 3D printing structures
Source: Nat Commun. 2024 Feb 21;15:1596. doi: 10.1038/s41467-024-45497-4 (PMC10882007; doi:10.1038/s41467-024-45497-4)
Supplement: Supplementary file 1 — Supplementary Information [file 41467_2024_45497_MOESM1_ESM.pdf]

Supplementary Information

Tunable afterglow for mechanical self-monitoring 3D printing structures

Rongjuan Huang[1,2,#], Yunfei He[1,#], Juan Wang[1], Jindou Zou[1], Hailan Wang[1], Haodong Sun[1], Yuxin Xiao[1], Dexin Zheng[3], Jiani Ma[3], Tao Yu[1,2,*] and Wei Huang[1,4,5,*]

[1]Frontiers Science Center for Flexible Electronics (FSCFE) and Xi'an Institute of Flexible Electronics (IFE), Northwestern Polytechnical University, 127 West Youyi Road, Xi'an 710072, China

[2]Key Laboratory of Flexible Electronics of Zhejiang Province, Ningbo Institute of Northwestern Polytechnical University, 218 Qingyi Road, Ningbo 315103, China

[3]Key Laboratory of Applied Surface and Colloid Chemistry, Ministry of Education, School of Chemistry and Chemistry Engineering, Shaanxi Normal University, Xi'an 710119, China

[4]Key Laboratory of Flexible Electronics (KLOFE) & Institute of Advanced Materials (IAM), Nanjing Tech University (Nanjing Tech), 30 South Puzhu Road, Nanjing 211816, China

[5]State Key Laboratory of Organic Electronics and Information Displays & Jiangsu Key Laboratory for Biosensors, Institute of Advanced Materials (IAM), Nanjing University of Posts and Telecommunications, 9 Wenyuan Road, Nanjing 210023, China

Address correspondence: iamtyu@nwpu.edu.cn; vc@nwpu.edu.cn

S1. Supplementary Discussion 2
- 1.1 Syntheses and Characterizations 2
- 1.2 Thermal Analyses 16
- 1.3 Photophysical Characterizations 19
- 1.4 Theoretical Calculations 32
- 1.5 3D printing structures 37

S2. Supplementary References 48

S1. Supplementary Discussion

1.1 Syntheses and Characterizations

All reagents and solvents were purchased from commercial sources and used without further purification. Manipulations were performed under normal atmosphere unless specially noted.

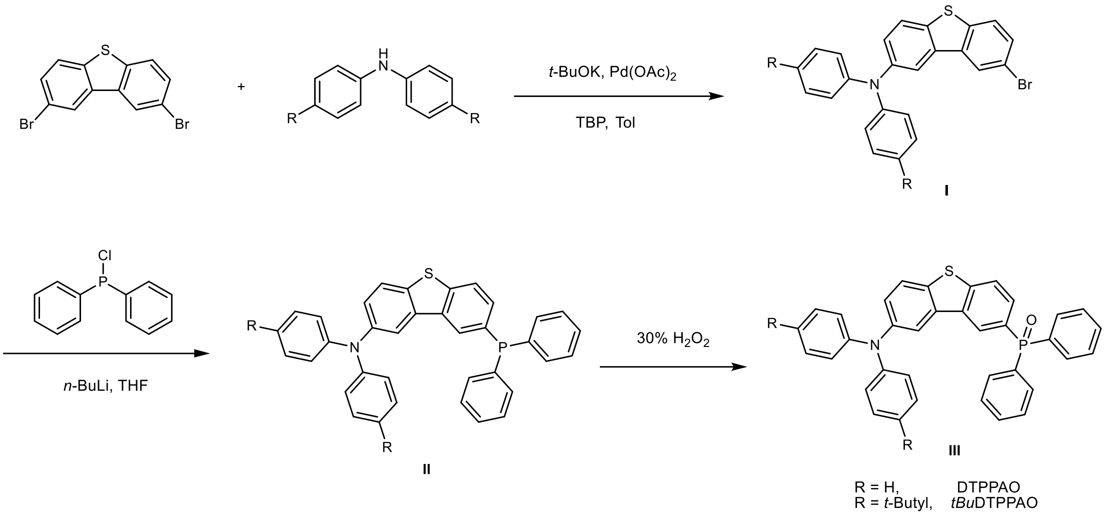

Supplementary Figure 1. Synthesis route of compounds DTPPAO and *tBu*DTPPAO.

Synthesis of (8-(diphenylamino)dibenzo[b,d]thiophen-2-yl)diphenylphosphine oxide (DTPPAO)

Synthesis of 8-bromo-N,N-diphenyldibenzo thiophen-2-amine (**I**): To a degassed toluene (Tol) (30 mL) containing 2,8-Dibromodibenzothiophene (1.00 g, 2.92 mmol), diphenylamine (0.444 g, 2.66 mmol) and *t*-BuOK (0.895 g, 7.97 mmol), Tri-tert-butylphosphorus (TBP) (0.050 g, 0.247 mmol) and $Pd(OAc)_2$ (50.0 mg, 0.220 mmol) were added under argon atmosphere. The mixture was heated to 115 °C for 16 h. After the reaction was completed, the mixture was poured into water and then extracted three times with dichloromethane. The organic layer was dried with anhydrous sodium sulfate after extraction. The solvent was removed by distillation under reduced pressure. The crude product was purified by silica gel (Dichloromethane and n-hexane (1:6)) column chromatography to obtain 8-bromo-N,N-diphenyldibenzothiophen-2-amine (0.215 g, 18.9%). ^{1}H NMR (500 MHz, DMSO-d_6): δ 8.53 (m, 1H), 8.19 (d, J = 2.2 Hz, 1H), 7.98 (dd, J = 8.6, 1.9 Hz, 2H), 7.63 (dd, J = 8.5, 2.0 Hz, 1H), 7.32 – 7.27 (m, 4H), 7.23 (dd, J = 8.6, 2.2 Hz, 1H), 7.05 – 7.00 (m, 6H). ^{13}C NMR (126 MHz, $CDCl_3$) δ 148.07, 145.65, 139.02, 137.18, 135.83, 134.42, 129.76, 129.52, 125.52, 124.79, 124.33, 124.07, 123.69, 122.97, 118.32, 117.62. HRMS (m/z): [M]+ calcd. for

C24H16BrNS, 430.026; found, 431.0163.

Synthesis of 8-(diphenylphosphaneyl)-N,N-diphenyldibenzo thiophen-2-amine (**II**): 8-Bromo-*N,N*-diphenyldibenzothiophen-2-amine (**I**) (0.500 g, 1.16 mmol) was dissolved in dry tetrahydrofuran (50 mL). *n*-BuLi (0.87 mL, 1.60 mol/L,1.39 mmol) was added in -78 °C. After 2 h, chlorodiphenylphosphine (0.307 g, 1.39 mmol) was added to the reaction solution at -78 °C. After 3 h, the reaction mixuture was gradually warmed to room temperature and stirred overnight. After the reaction was completed, the reaction mixture was addded to deionized water (50 mL) and extracted 3 times with dichloromethane. The organic layer was dried with anhydrous sodium sulfate after extraction. The solvent was removed by distillation under reduced pressure. The crude product was purified by silica gel (ethyl acetate and n-hexane (1:1)) column chromatography to obtain a yellow solid (0.152 g, 23.0%). ^{1}H NMR (500 MHz, DMSO-d_6): δ 8.07 (dd, J = 9.0, 1.5 Hz, 1H), 8.03 (d, J = 8.2 Hz, 1H), 7.97 – 7.94 (m, 1H), 7.83 (d, J = 2.2 Hz, 1H), 7.41 – 7.35 (m, 6H), 7.29 (dd, J = 8.5, 7.3 Hz, 4H), 7.26 – 7.19 (m, 6H), 7.07 – 7.00 (m, 6H). ^{13}C NMR (126 MHz, $CDCl_3$) δ 148.06, 145.52, 141.39, 137.46, 137.38, 136.47, 133.98, 133.98, 133.77, 133.61, 132.89, 132.80, 131.82, 131.72, 129.46, 128.86, 128.70, 128.64, 128.32, 128.07, 125.02, 124.05, 123.59, 123.25, 123.21, 122.89, 117.55. HRMS (m/z): [M]+ calcd. for C36H26NPS, 536.1596; found, 536.1595.

Synthesis of DTPPAO (**III**): The 8-(diphenylphosphaneyl)-N,N-diphenyldibenzo thiophen-2-amine (**II**) (0.152 g, 0.285 mmol) was dissolved in THF (10 mL), H_2O_2 (1 mL 30%) was added. The reaction was completed after half an hour. The reaction solution is dried by distillation under reduced pressure. The reaction mixture was extracted 3 times with deionized water and dichloromethane. The organic layer was dried with anhydrous sodium sulfate after extraction. Removing solvent from organic layer through vacuum distillation to obtain a yellow solid (0.143 g, 91.2%). ^{1}H NMR (500 MHz, DMSO-d_6): δ 8.42 (dd, J = 12.3, 1.5 Hz, 1H), 8.18 (dd, J = 8.3, 2.3 Hz, 1H), 7.99 (dd, J = 13.6, 5.4 Hz, 2H), 7.68 – 7.59 (m, 7H), 7.54 (dd, J = 7.6, 2.7 Hz, 4H), 7.34 – 7.27 (m, 4H), 7.25 (dd, J = 8.6, 2.2 Hz, 1H), 7.04 (d, J = 8.7 Hz, 6H). ^{13}C NMR (126 MHz, $CDCl_3$) δ 148.00, 145.94, 144.47, 144.44, 136.30, 135.74, 135.64, 133.81, 133.23, 132.40, 132.31, 132.23, 132.18, 132.16, 129.54, 129.36, 129.26, 128.76, 128.66, 128.50, 127.66, 126.24, 126.17, 125.55, 124.08, 123.58, 123.05, 123.02, 122.91, 117.98. HRMS (m/z): [M]+ calcd. for C36H26NOPS, 552.1506; found, 552.1547.

Synthesis of *tBu*DTPPAO

Following the synthesis of DTPPAO, *tBu*DTPPAO was obtained as a yellow solid (yield: 23.6%). ^{1}H NMR (500 MHz, DMSd$_6$): δ 8.41 (d, J = 11.0 Hz, 1H), 8.18 (dd, J = 8.3, 2.3 Hz, 1H), 7.96 (dd, J = 5.4, 3.2 Hz, 2H), 7.67 – 7.59 (m, 7H), 7.52 (m, 4H), 7.31 (d, J = 8.7 Hz, 4H), 7.18 (dd, J = 8.7, 2.2 Hz, 1H), 6.96 (d, J = 8.7 Hz, 4H), 1.27 (s, 18H). ^{13}C NMR (126 MHz, $CDCl^3$) δ 146.25, 145.60, 145.41, 144.51, 144.49, 136.23, 135.90, 135.79, 133.26, 132.43, 132.32, 132.24, 132.17, 132.15, 129.28, 129.18, 128.75, 128.65, 128.35, 127.51, 126.30, 125.54, 123.45, 117.66, 34.42, 31.59. HRMS (m/z): [M]+ calcd. for C44H42NOPS, 663.2725; found, 663.2717.

*TBu*DTPPAO (**I**)**:** ^{1}H NMR (500 MHz, DMSO-d$_6$): δ 8.54 (d, J = 1.9 Hz, 1H), 8.17 (d, J = 2.2 Hz, 1H), 7.99 – 7.93 (m, 2H), 7.63 (dd, J = 8.5, 2.0 Hz, 1H), 7.32 – 7.29 (m, 4H), 7.17 (dd, J = 8.6, 2.2 Hz, 1H), 6.95 – 6.92 (m, 4H), 1.27 (s, 18H). ^{13}C NMR (126 MHz, $CDCl_3$) δ 145.95, 145.65, 145.43, 139.04, 137.32, 135.74, 133.79, 129.62, 126.29, 125.32, 124.82, 124.32, 123.50, 118.21, 117.10, 34.43, 31.60. HRMS (m/z): [M]+ calcd. for C32H32BrNS, 542.1510; found, 543.1422.

*TBu*DTPPAO (**II**): ^{1}H NMR (500 MHz, DMSO-d$_6$) δ 8.09 (dd, J = 8.9, 1.5 Hz, 1H), 8.03 (d, J = 8.1 Hz, 1H), 7.92 (d, J = 8.6 Hz, 1H), 7.84 (d, J = 2.2 Hz, 1H), 7.37 (d, J = 5.5 Hz, 6H), 7.29 (d, J = 8.7 Hz, 4H), 7.25 (m, 5H), 7.14 (dd, J = 8.6, 2.2 Hz, 1H), 6.93 (d, J = 8.7 Hz, 4H), 1.27 (s, 18H). ^{13}C NMR (126 MHz, $CDCl_3$) δ 145.79, 145.49, 145.45, 141.46, 137.51, 137.43, 136.39, 135.82, 135.74, 133.76, 133.61, 133.45, 132.69, 132.60, 131.72, 131.63, 128.85, 128.70, 128.65, 128.41, 128.16, 126.21, 125.02, 123.46, 123.41, 123.27, 123.22, 117.20, 34.41, 31.73, 31.61. HRMS (m/z): [M]+ calcd. for C44H42NPS, 648.2848; found, 648.2847.

Synthesis of DTPA

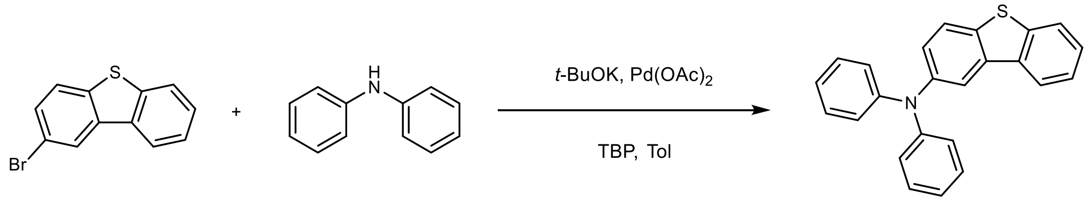

DTPA

Supplementary Figure 2. Synthesis route of compounds DTPA.

2-Bromodibenzothiophene (1.00 g, 3.80 mmol) and diphenylamine (0.71 g, 4.18 mmol) dissolved in toluene (Tol) (30 mL) were added to the flask under argon atmosphere. Stir and bubble for 20 min, then add *t*-BuOK (1.28 g, 11.4 mmol), $Pd(OAc)_2$ (17.96 mg, 0.08 mmol), and Tri-tert-butylphosphorus (TBP) (0.41 g, 2.00 mmol). The reaction was heated to 110°C and refluxed for 16 h. After the reaction was completed, the reaction

solution was cooled to room temperature and spun-dried. The organic phase was extracted three times with dichloromethane and deionized water and collected. The organic phase was then dried with Na_2SO_4, spun-dried under reduced pressure, and the crude product was separated and purified by column chromatography (dichloromethane and hexane (1:5)) to obtain transparent colorless crystals (1.18 g, 88.06%). ^1H NMR (500 MHz, $CDCl_3$): δ 7.95 (d, J = 7.9 Hz, 1H), 7.89 (d, J = 1.8 Hz, 1H), 7.82 (d, J = 7.8 Hz, 1H), 7.72 (d, J = 8.6 Hz, 1H), 7.43 - 7.35 (m, 2H), 7.27 (s, 1H), 7.26 - 7.21 (m, 4H), 7.16 - 7.08 (m, 4H), 7.02 (dd, J = 10.5, 4.2 Hz, 2H).

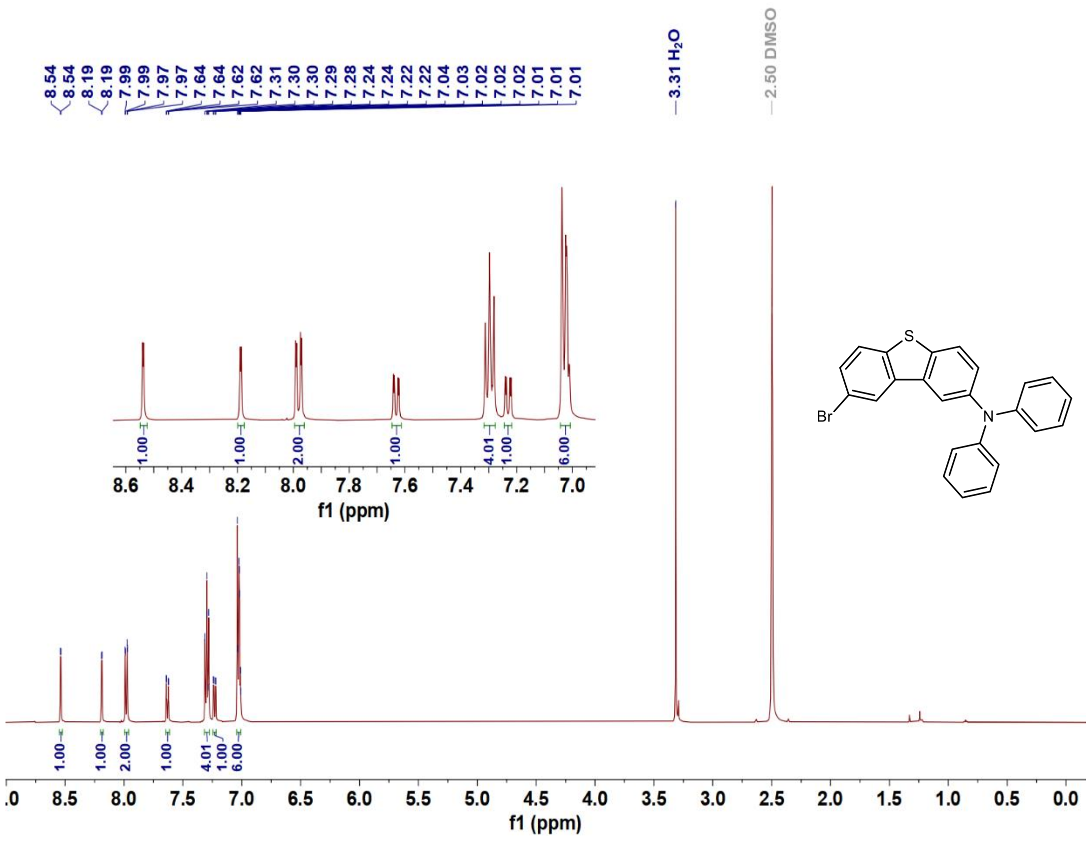

Supplementary Figure 3. ^1H NMR spectrum of **I** (DTPPAO).

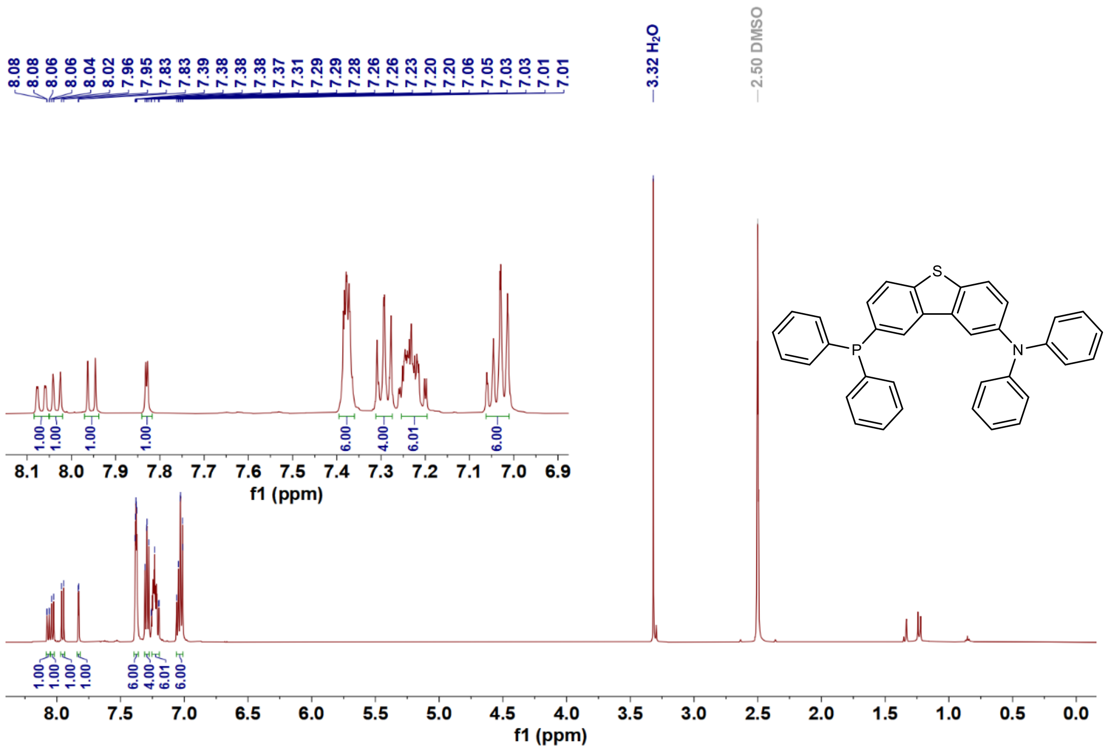

Supplementary Figure 4. ^{1}H NMR spectrum of **II** (DTPPAO).

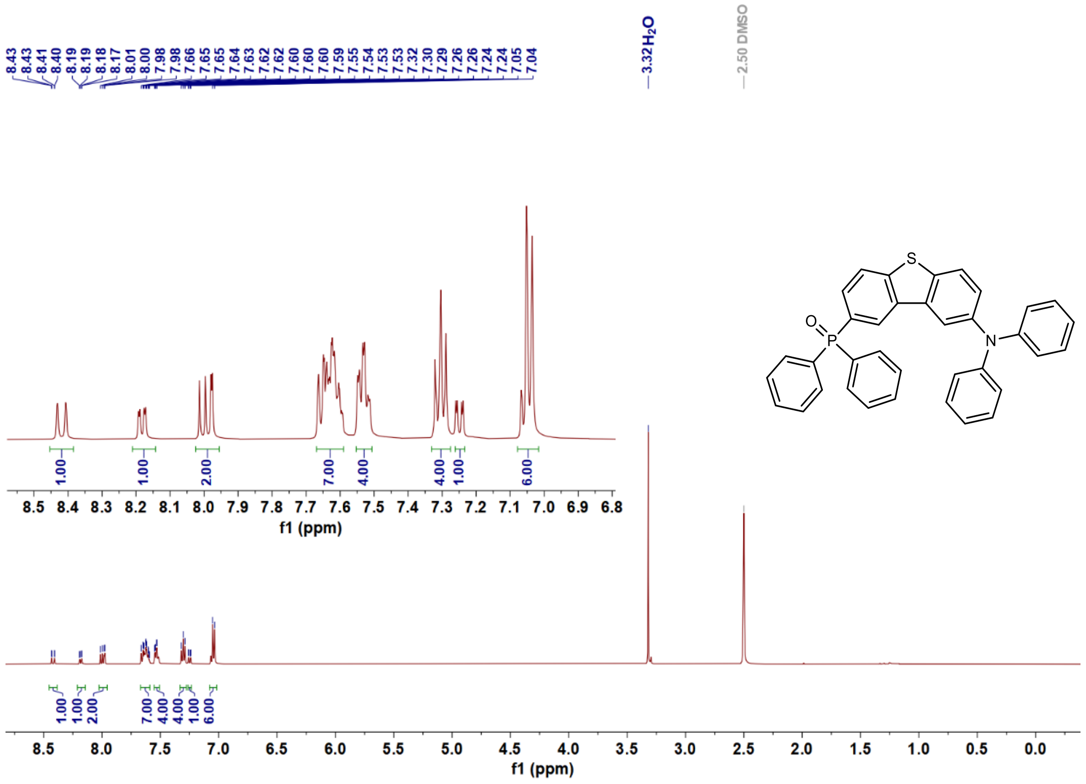

Supplementary Figure 5. ^{1}H NMR spectrum of DTPPAO.

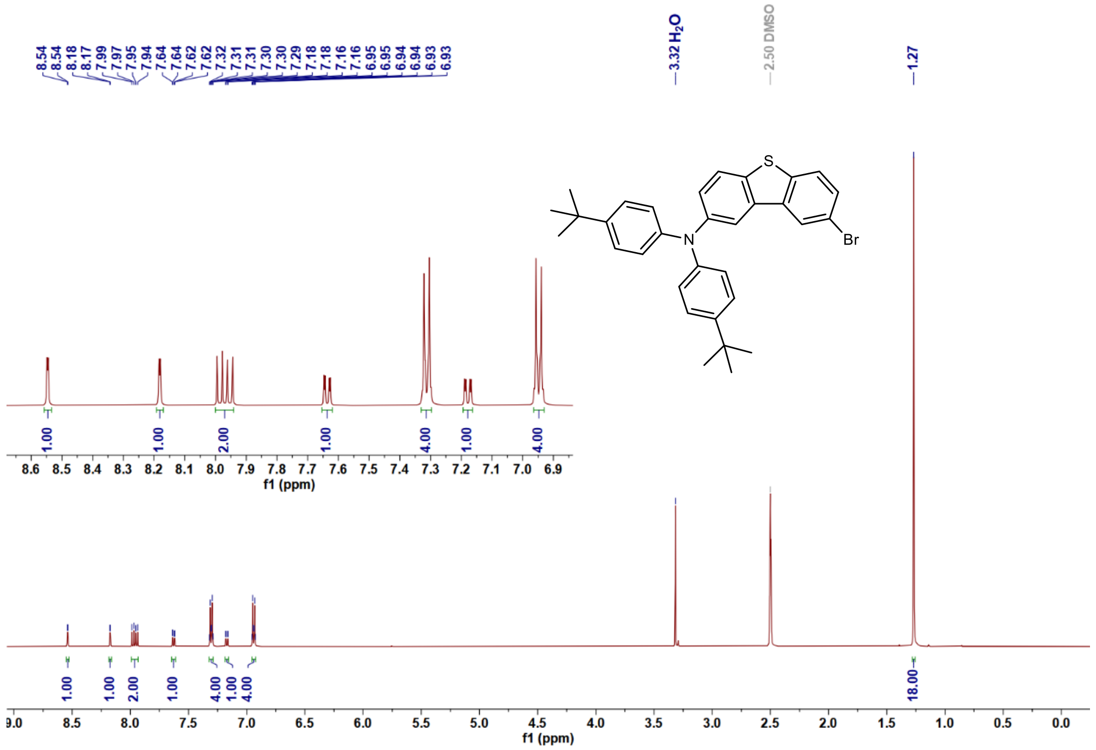

Supplementary Figure 6. ^{1}H NMR spectrum of **I** (*tBu*DTPPAO).

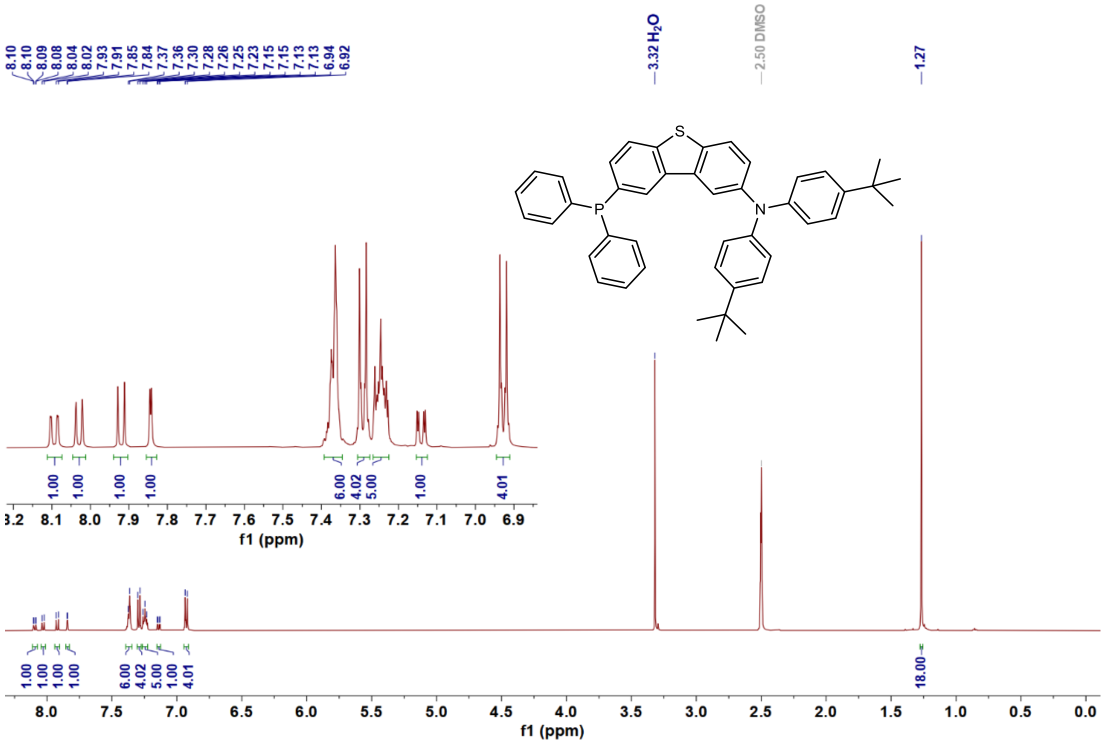

Supplementary Figure 7. ^{1}H NMR spectrum of **II** (*tBu*DTPPAO).

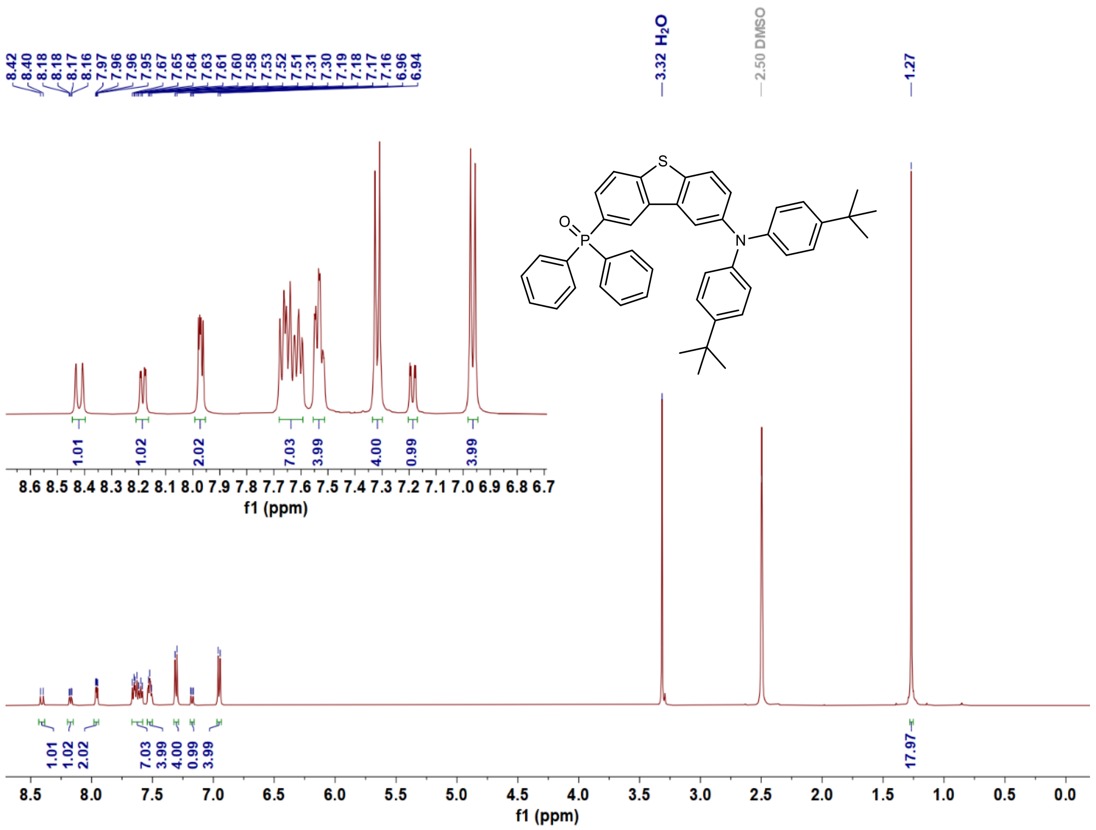

Supplementary Figure 8. ^1H NMR spectrum of *tBu*DTPPAO.

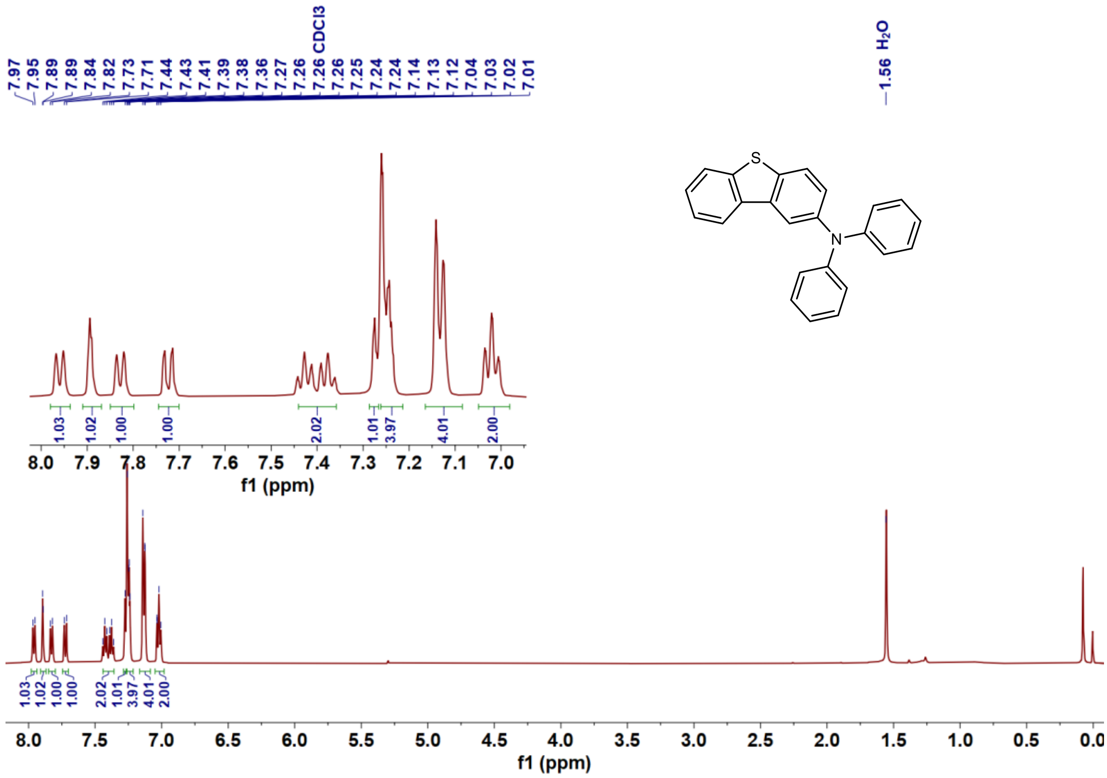

Supplementary Figure 9. ^1H NMR spectrum of DTPA.

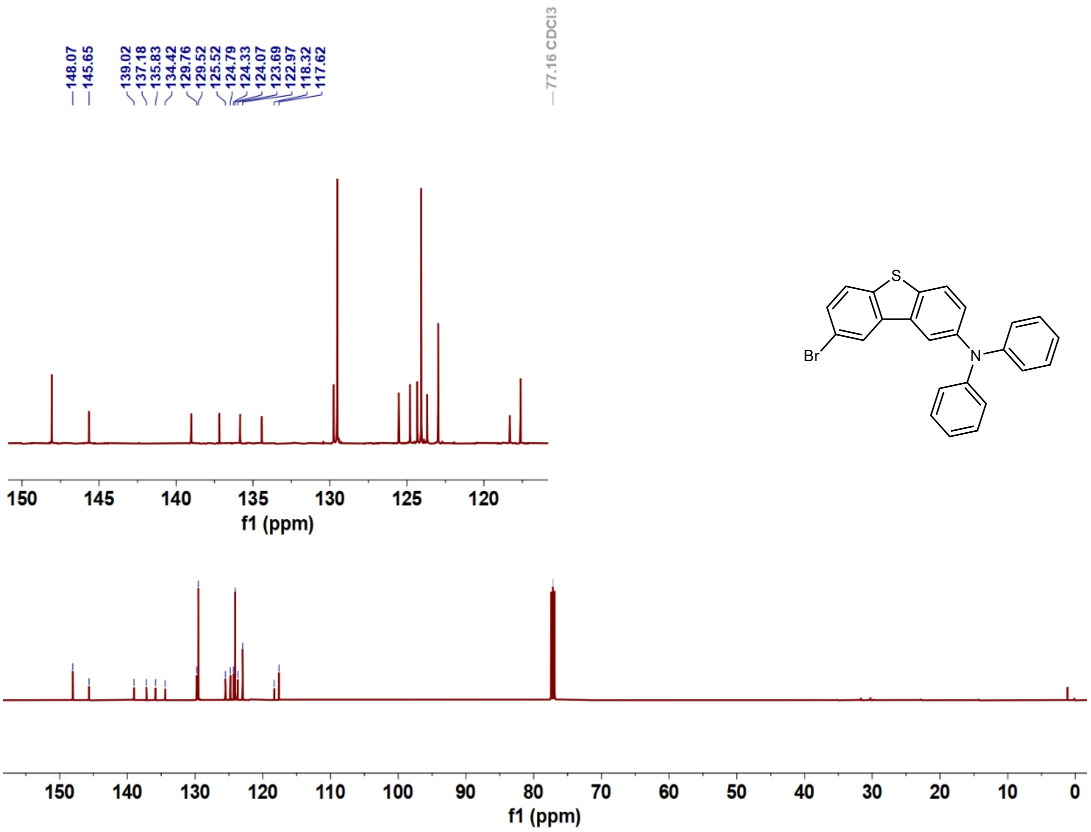

Supplementary Figure 10. ^{13}C NMR spectrum of **I** (DTPPAO).

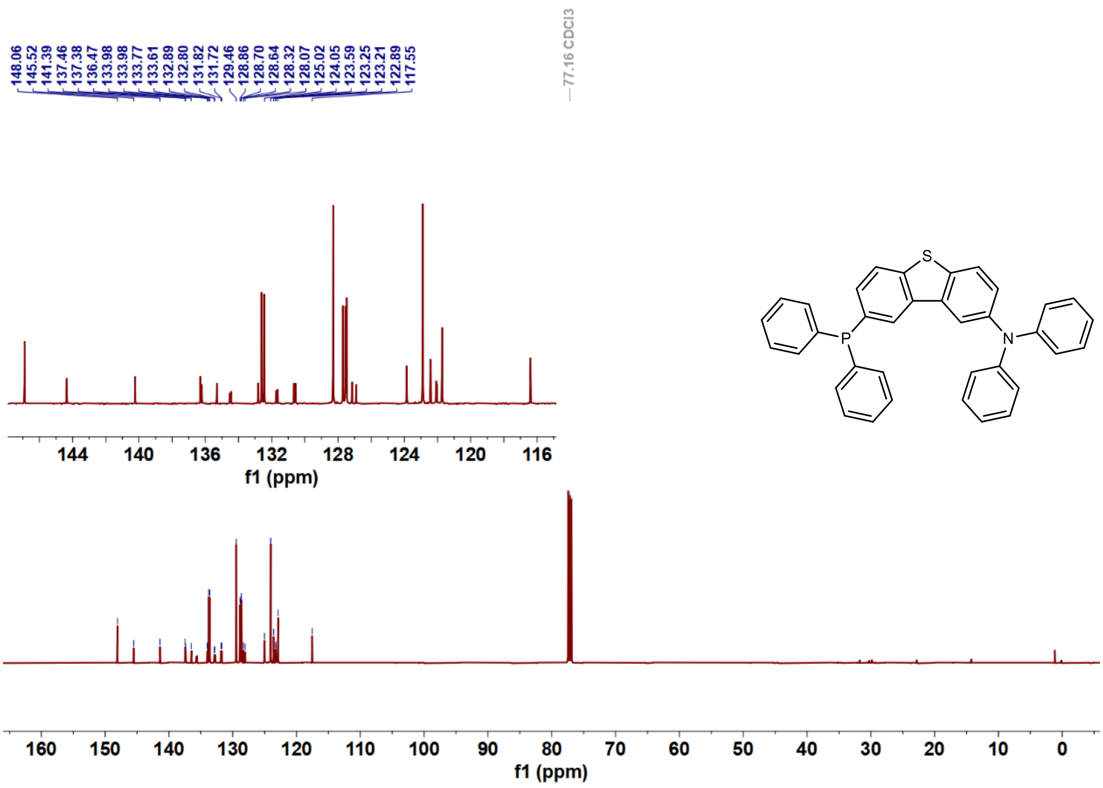

Supplementary Figure 11. ^{13}C NMR spectrum of **II** (DTPPAO).

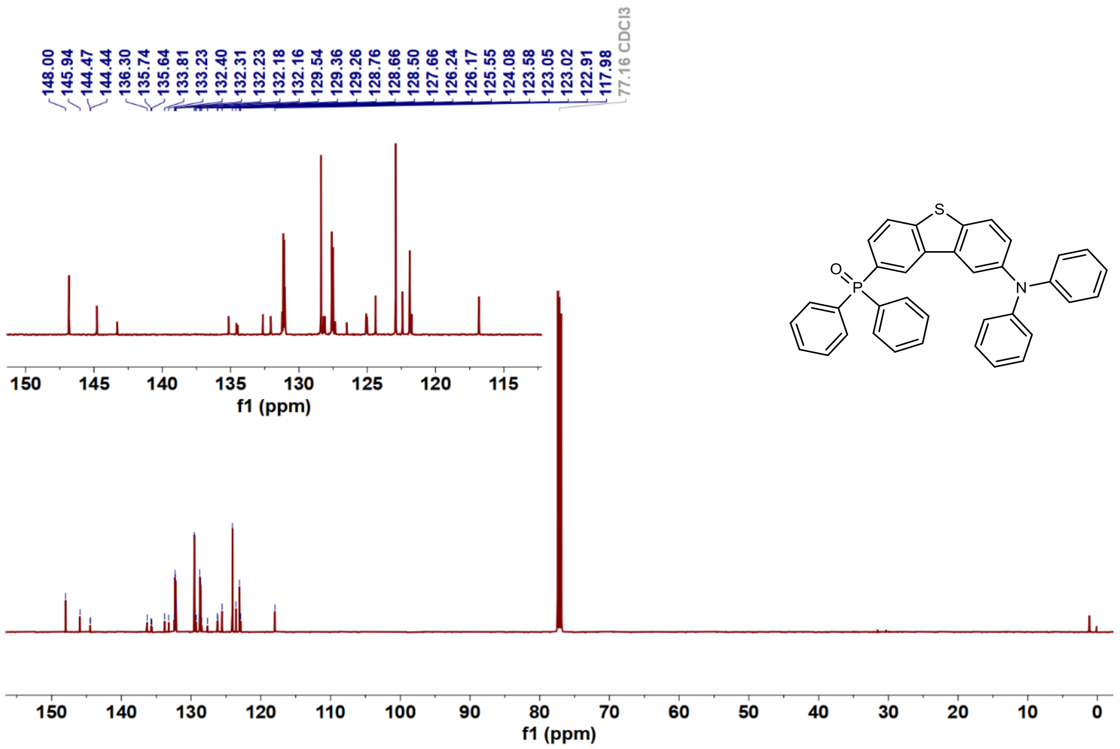

Supplementary Figure 12. ^{13}C NMR spectrum of DTPPAO.

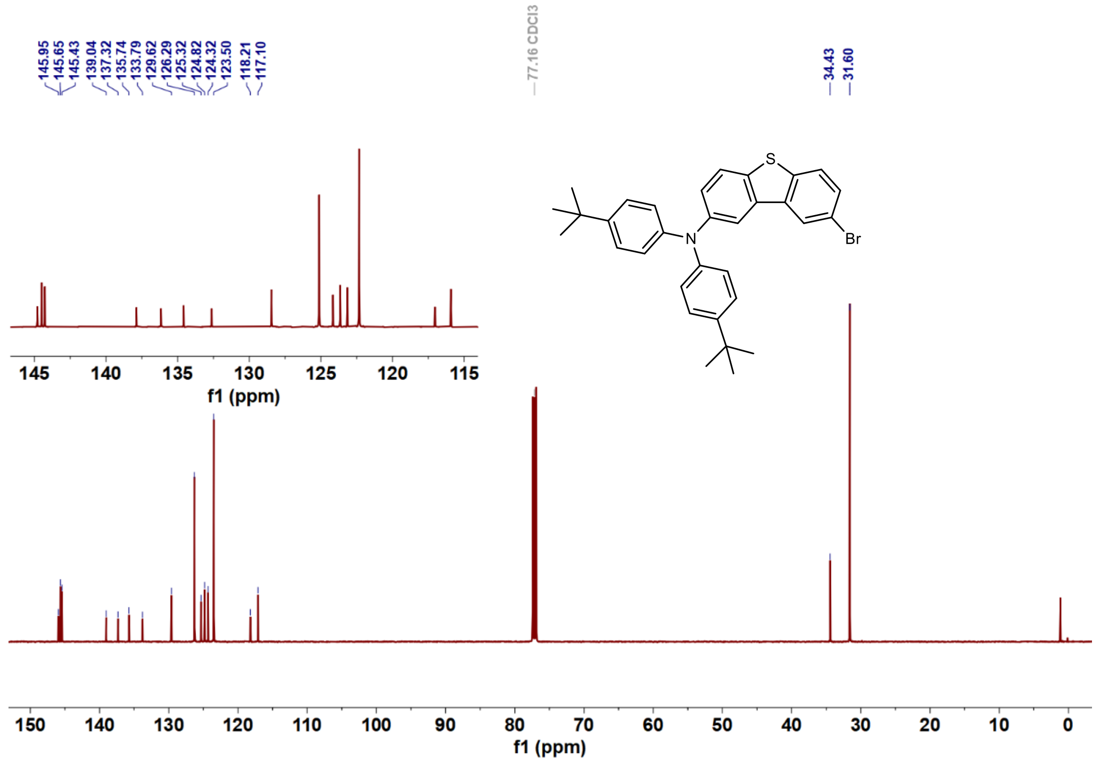

Supplementary Figure 13. ^{13}C NMR spectrum of **I** (*tBu*DTPPAO).

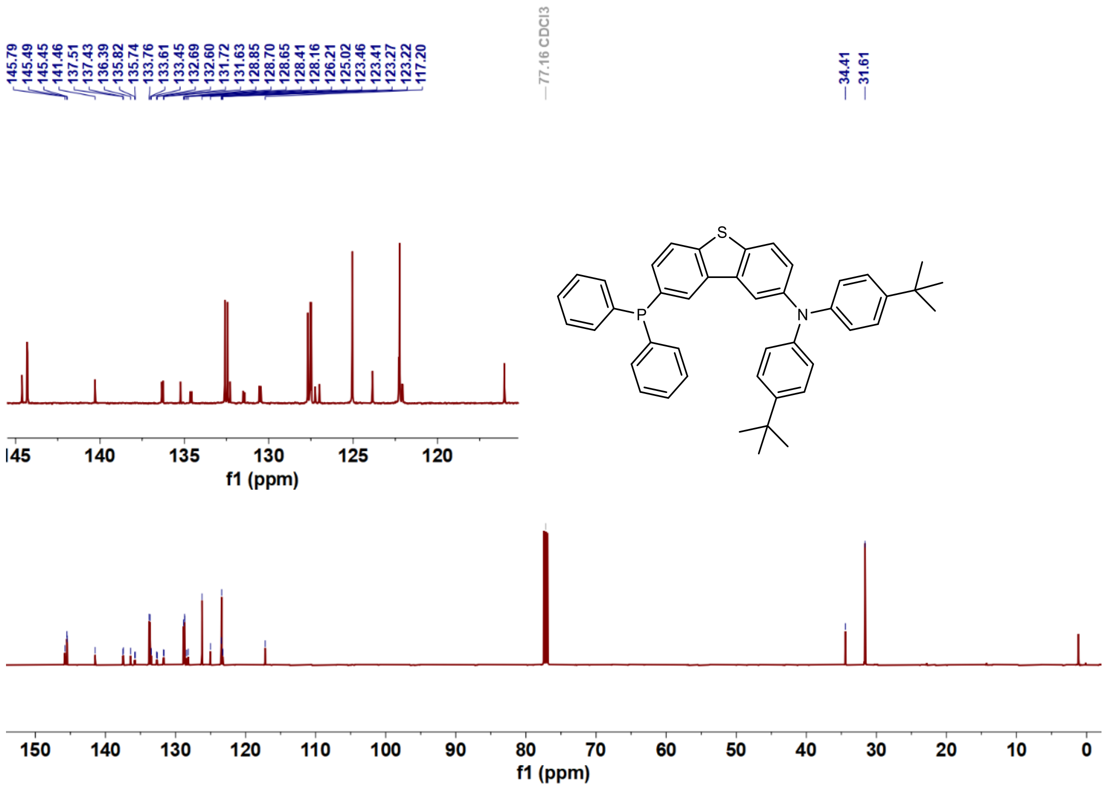

Supplementary Figure 14. ^{13}C NMR spectrum of **II** (*tBu*DTPPAO).

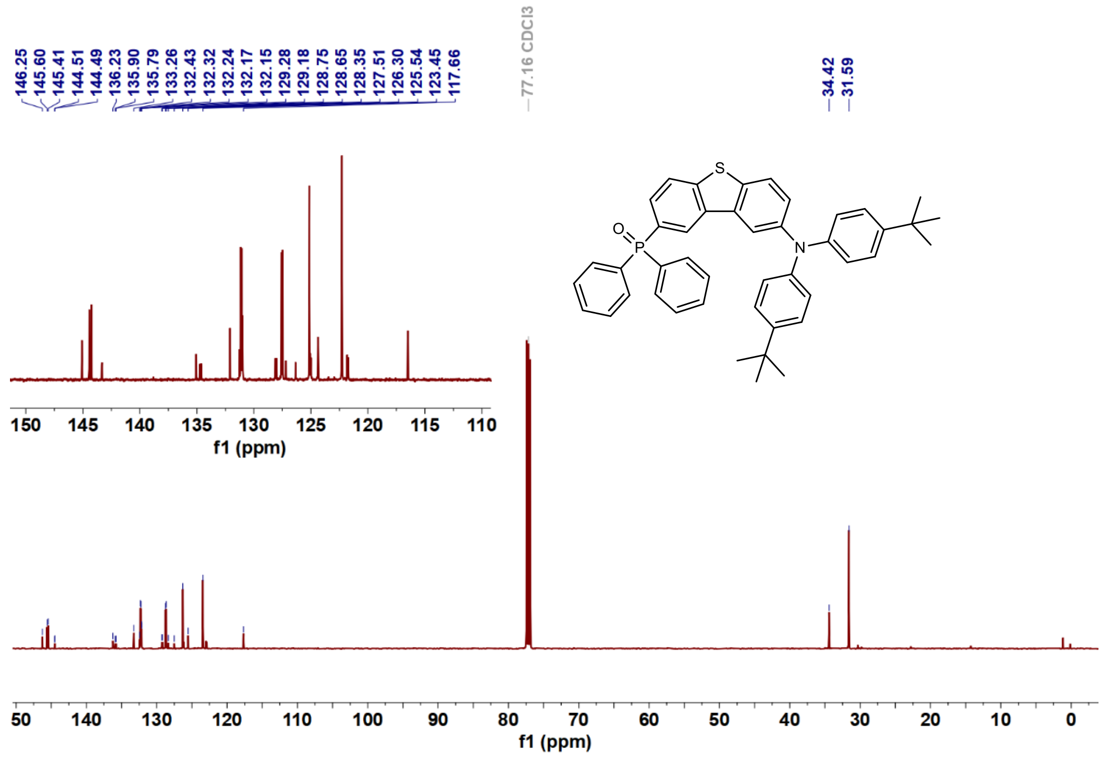

Supplementary Figure 15. ^{13}C NMR spectrum of *tBu*DTPPAO.

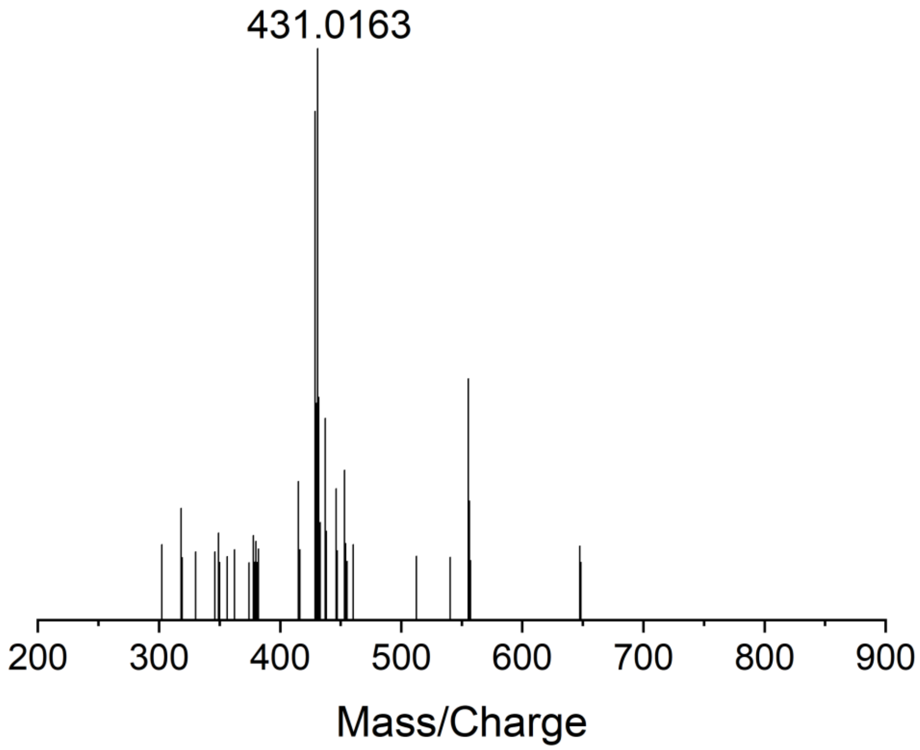

Supplementary Figure 16. High resolution mass spectrum of **I** (DTPPAO).

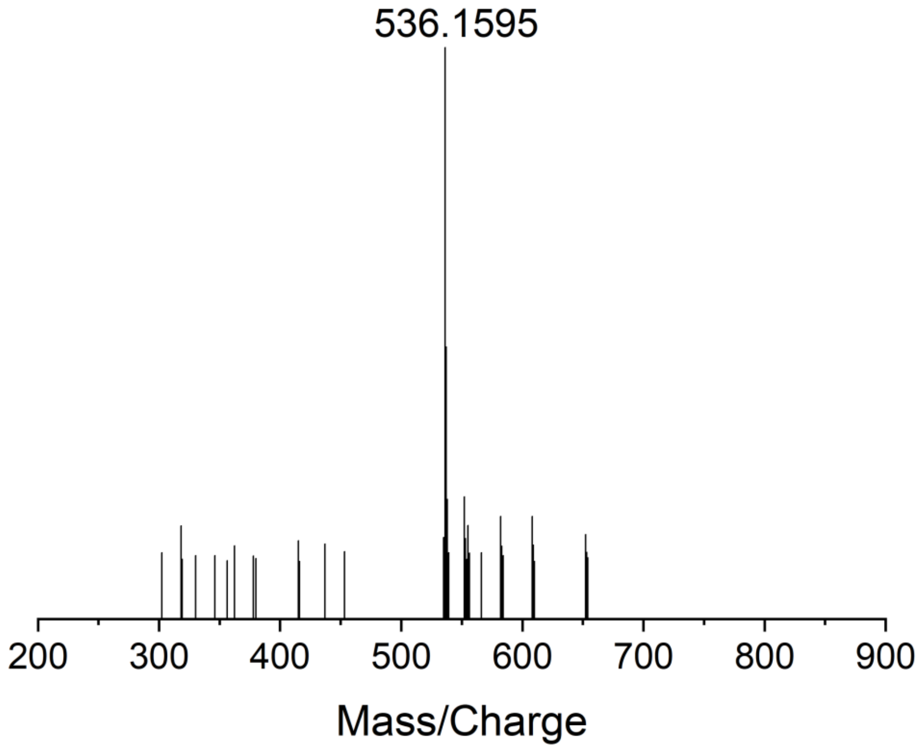

Supplementary Figure 17. High resolution mass spectrum of **II** (DTPPAO).

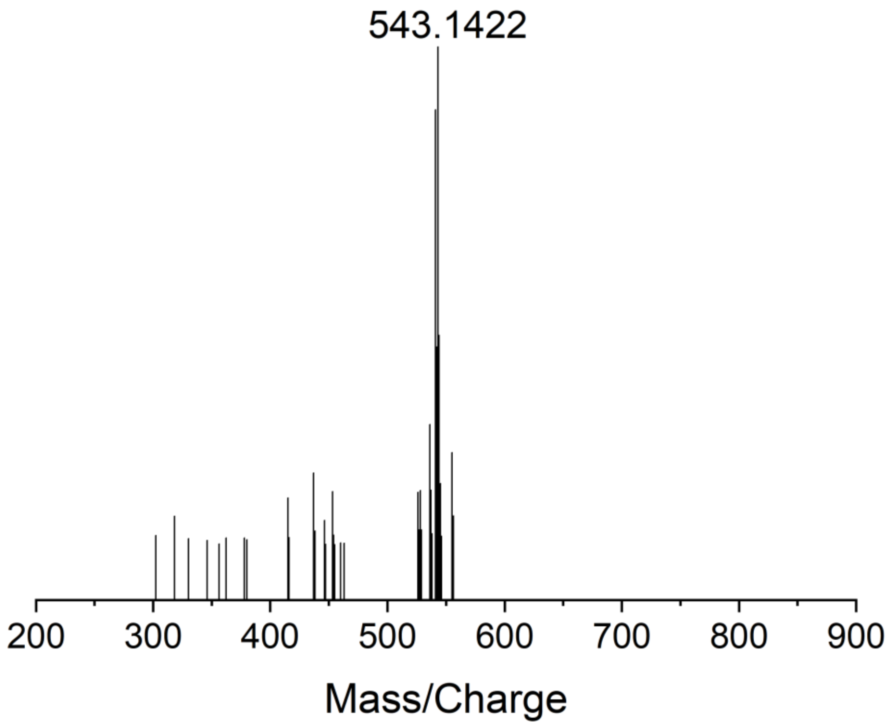

Supplementary Figure 18. High resolution mass spectrum of **I** (*tBu*DTPPAO).

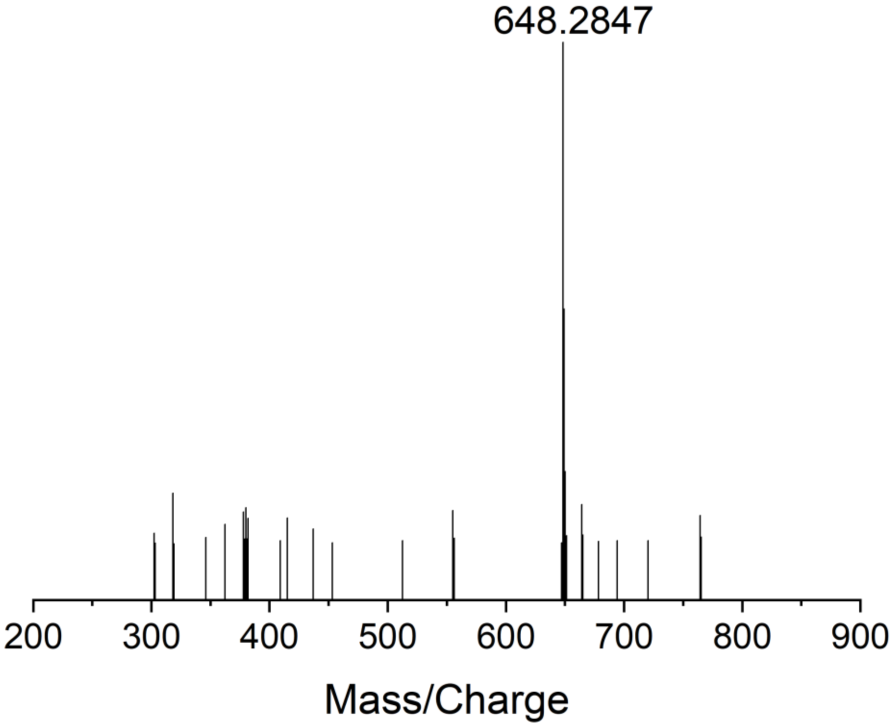

Supplementary Figure 19. High resolution mass spectrum of **II** (*tBu*DTPPAO).

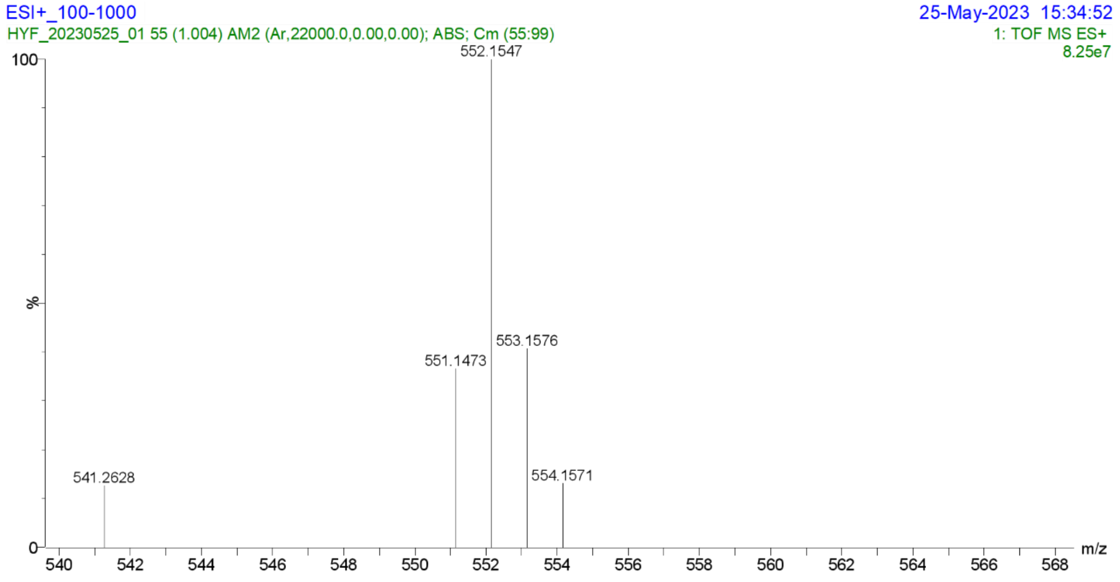

Supplementary Figure 20. High resolution mass spectrum of DTPPAO.

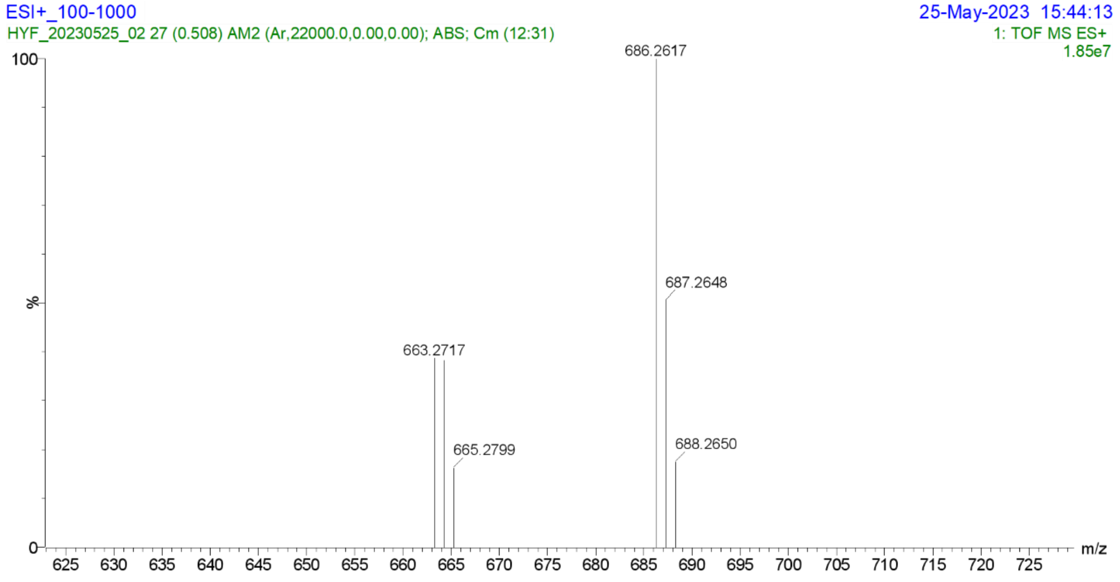

Supplementary Figure 21. High resolution mass spectrum of *tBu*DTPPAO.

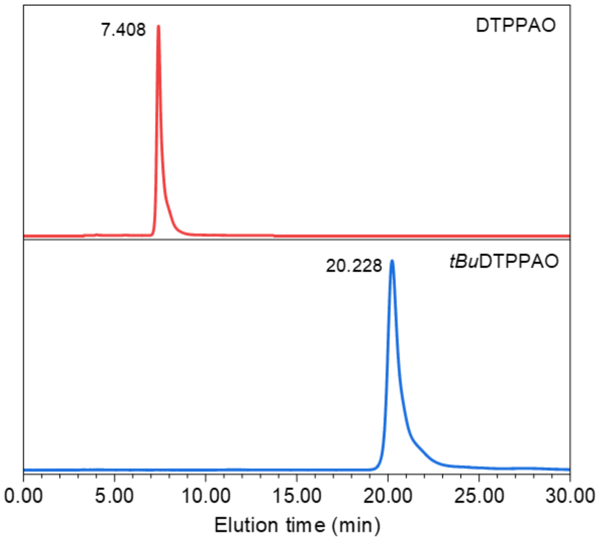

Supplementary Figure 22. High performance liquid chromatography (HPLC) spectra of DTPPAO and *tBu*DTPPAO in methanol solution.

Supplementary Table 1. Elemental analyses of DTPPAO and *tBu*DTPPAO.

**Element content (%)**		**C**	**H**	**N**
**DTPPAO**	Theoretical value	78.38	4.75	2.54
	Experimental value	78.553	4.695	2.543
***tBu*DTPPAO**	Theoretical value	79.61	6.38	2.11
	Experimental value	79.875	6.409	2.043

1.2 Thermal Analyses

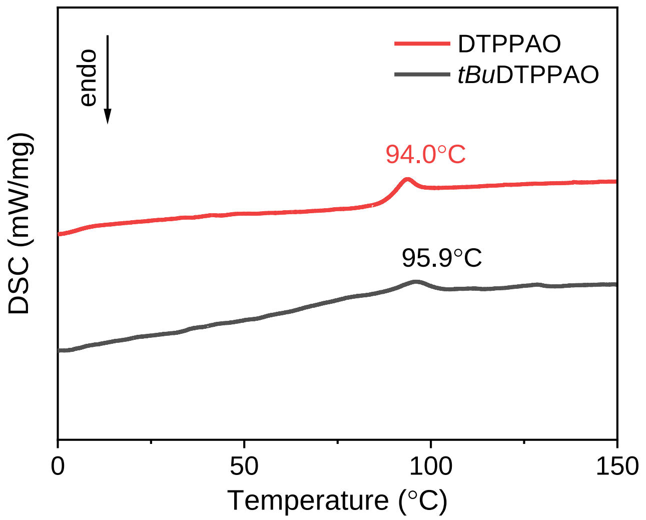

Supplementary Figure 23. DSC curves of DTPPAO and *tBu*DTPPAO under N_2 atmosphere at heating rate of 10°C/min.

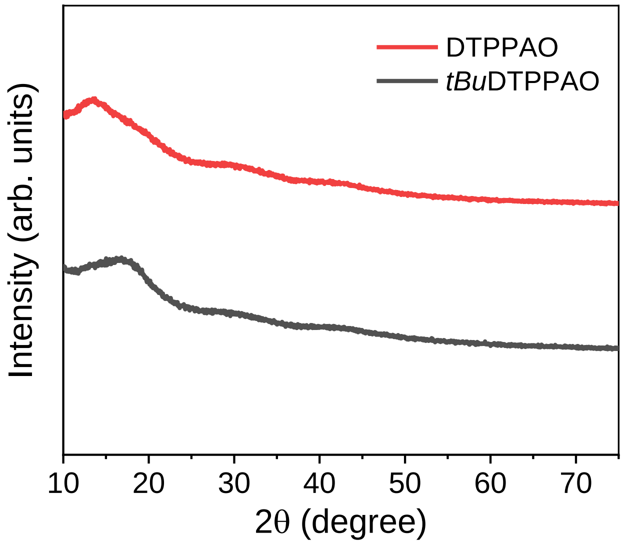

Supplementary Figure 24. X-ray powder diffraction of DTPPAO and *tBu*DTPPAO.

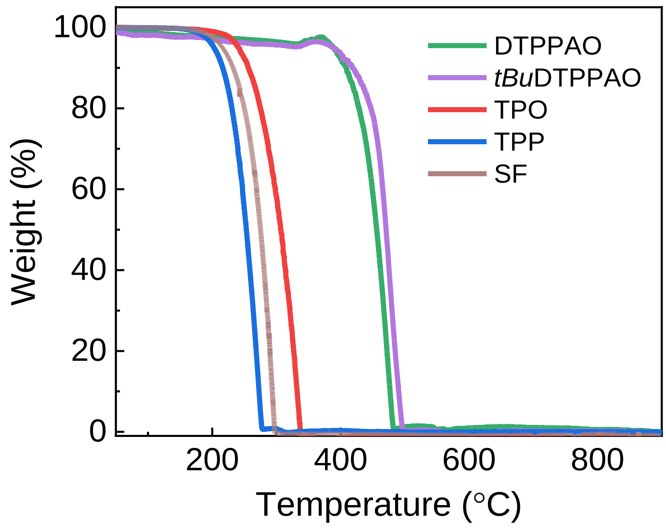

Supplementary Figure 25. Thermogravimetry analyses (TGA) curves of guest (DTPPAO and *tBu*DTPPAO) and host matrices (TPO, TPP and SF) under N_2 atmosphere at 20°C/min heating rate.

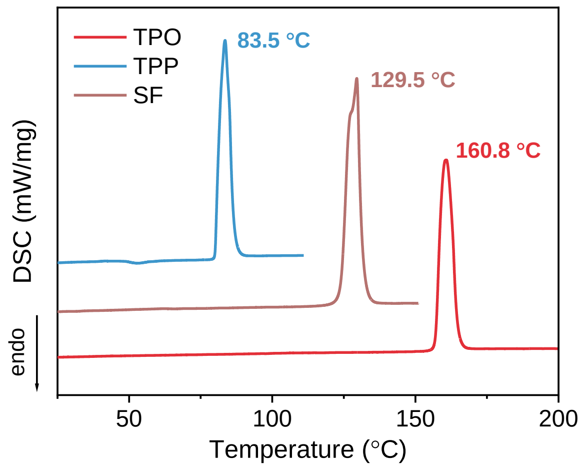

Supplementary Figure 26. Differential scanning calorimeter (DSC) curves of host molecules (TPO, TPP and SF) under N_2 atmosphere at 10°C/min heating rate.

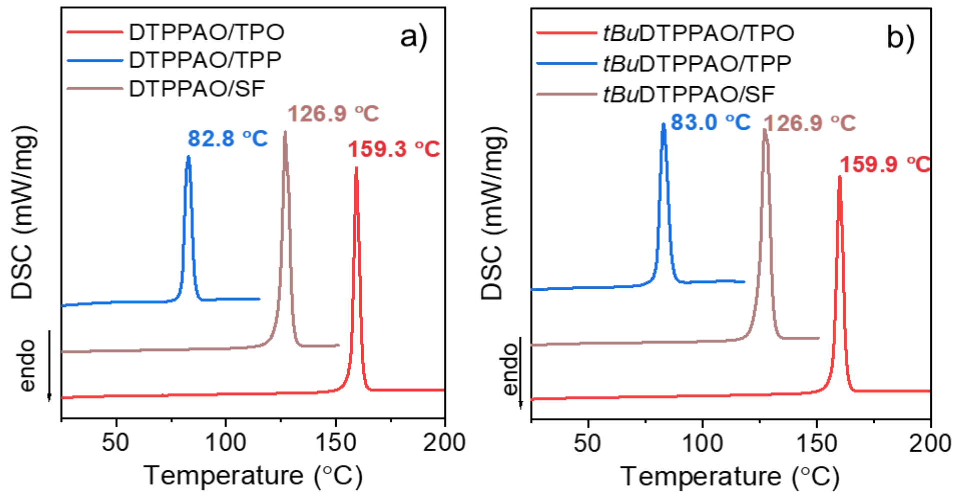

Supplementary Figure 27. Differential scanning calorimeter (DSC) curves of a) DTPPAO/host and b) *tBu*DTPPAO/host doping systems under N_2 atmosphere at 10°C/min heating rate.

Supplementary Table 2. Thermal properties of crystalline powders of guest (DTPPAO and *tBu*DTPPAO), host materials (TPO, TPP and SF) and host/guest doping systems.

	**DTPPAO**	***tBu*DTPPAO**	**TPO**	**TPP**	**SF**
**T_m (°C)**	94.0	95.9	160.8	83.5	129.5
**T_d (°C)**	388.2	388.3	239.5	202.2	213.6

*melting temperature (T_m) and decomposition temperatures (T_d) of 5% weight loss under N_2 atmosphere at 10°C/min and 20°C/min heating rate.

The syntheses and structural characterizations of DTPPAO and *tBu*DTPPAO are presented above in details. Their structures were unambiguously established by a combination of ^{1}H NMR, ^{13}C NMR spectroscopy, high-resolution mass spectrometry, element analysis (EA) and high-performance liquid chromatography (HPLC). The detailed characterizations of their respective intermediates **I** and **II** were also performed. To avoid any possible interference of impurities, strict purification procedures including column chromatography and recrystallization for three times were used. DSC (differential scanning calorimeter) and TGA (thermogravimetry analyses) studies were performed for the guest, host and doping systems. The melting points (T_m) of DTPPAO and *tBu*DTPPAO are 94.0°C and 95.9°C, respectively, indicating that both compounds can be fabricated by melt-casting method. The guest/host systems show different melting points varied with the host materials.

1.3 Photophysical Characterizations

Supplementary Table 3. Energy levels and photophysical properties in 2-MeTHF and PMMA matrix.

	HOMO/LUMO (eV)[a]	$\lambda_{abs.}$ (nm)[b]	S_1/T_1 (eV)[c]	ΔE_{ST} (eV)[d]	$\lambda_{PL}/\lambda_{PH}$ (nm)[e]	S_1/T_1 (eV)[f]	ΔE_{ST} (eV)[g]
**DTPPAO**	-5.38/-1.55	301, 372	3.16/2.92	0.24	417/466	3.24/2.90	0.34
***tBu*DTPPAO**	-5.21/-1.50	301, 375	3.11/2.88	0.23	432/473	3.15/2.86	0.29

[a]Energy levels calculated from the DFT. [b]The peaks of absorption spectra in 2-MeTHF solution. Calculated from the onsets of the fluorescence (RT) and phosphorescence spectra (77 K) [c]in 2-MeTHF and [f]in doped PMMA films. Experimentally determined singlet–triplet energy splitting in [d]2-MeTHF solution and [g]doped PMMA film. [e]The peaks of fluorescence (RT) and phosphorescence spectra (77 K) in PMMA films.

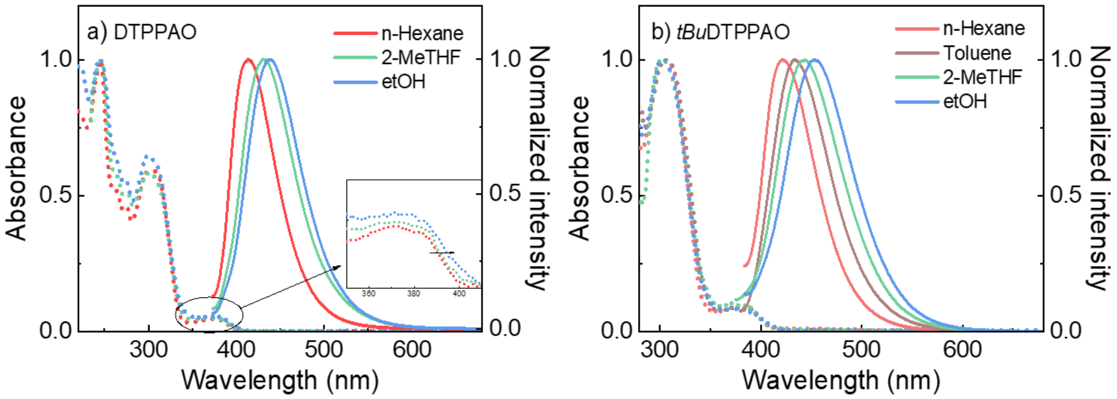

Supplementary Figure 28. Normalized UV-vis absorption and emission spectra of a) DTPPAO and b) *tBu*DTPPAO in solvents with different polarity (1×10^{-5} M): n-Hexane, 2-methyl tetrahydrofuran (2-MeTHF), toluene and etOH (ethanol) at room temperature. Inset in a) presents the enlarged part of the circle.

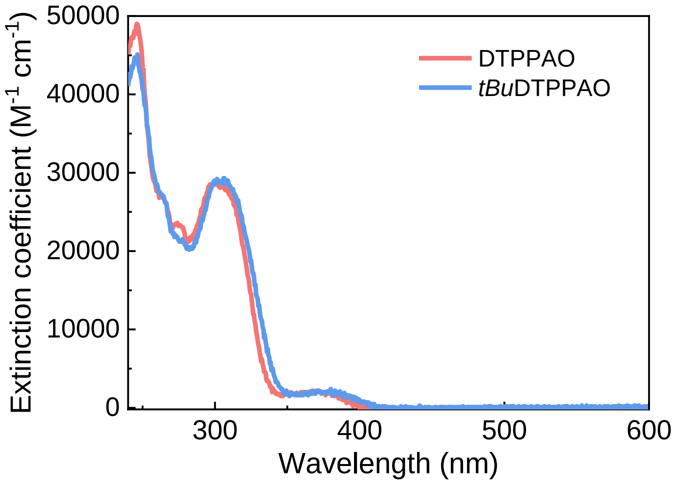

Supplementary Figure 29. Molar extinction coefficient of DTPPAO and *tBu*DTPPAO in 2-methyltetrahydrofuran solution (2-MeTHF, 10^{-5} mol/L) at room temperature.

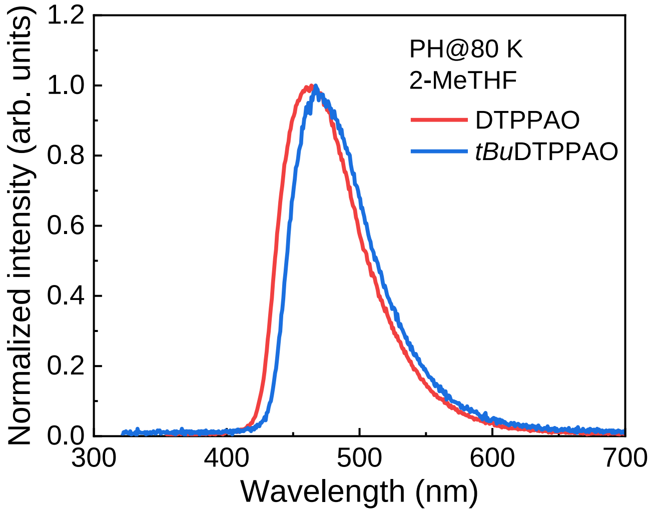

Supplementary Figure 30. Phosphorescence spectra of DTPPAO and *tBu*DTPPAO in 2-methyltetrahydrofuran solution (2-MeTHF, 10^{-5} mol/L) collected at 80 K.

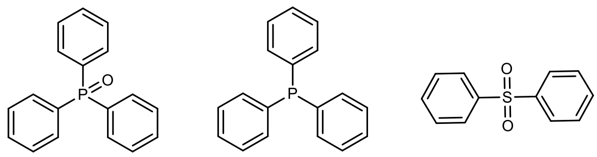

TPO TPP SF

Supplementary Figure 31. Molecular structures of small molecule host matrices: triphenylphosphine oxide (TPO), triphenylphosphine (TPP) and diphenyl-sulfone (SF).

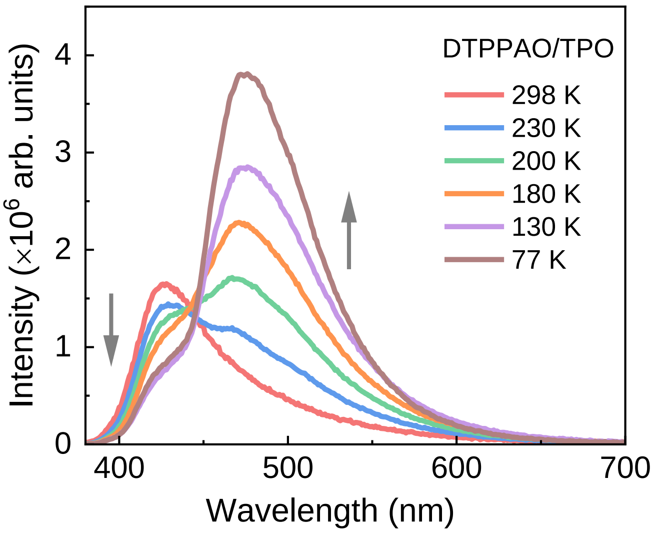

Supplementary Figure 32. Temperature-dependent emission spectra of 1 wt%DTPPAO:TPO.

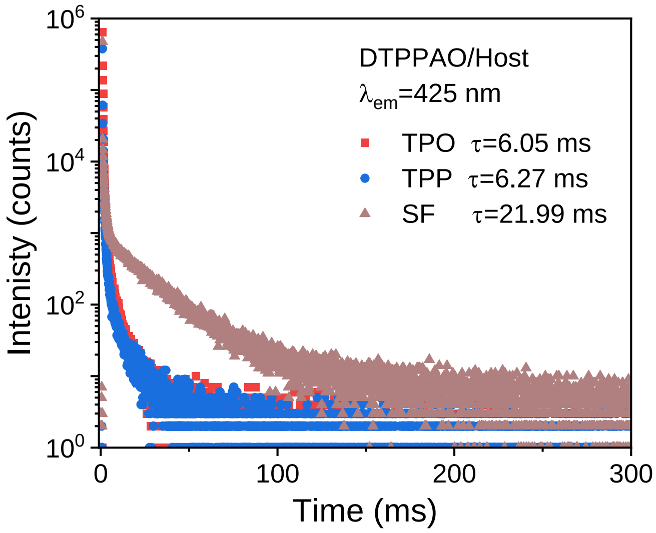

Supplementary Figure 33. Time-resolved decay curves of DTPPAO/host doping systems recorded at the fluorescence band of 425 nm at room temperature.

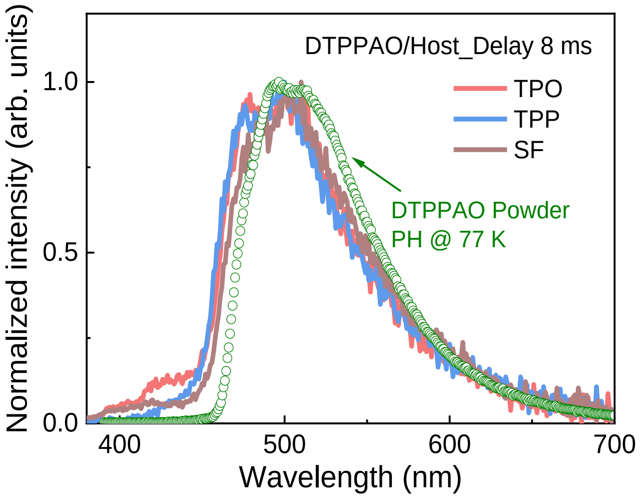

Supplementary Figure 34. Comparison of delayed spectra of DTPPAO in different host matrices at delay time of 8 ms (room temperature) and phosphorescence spectrum of DTPPAO powder (77 K).

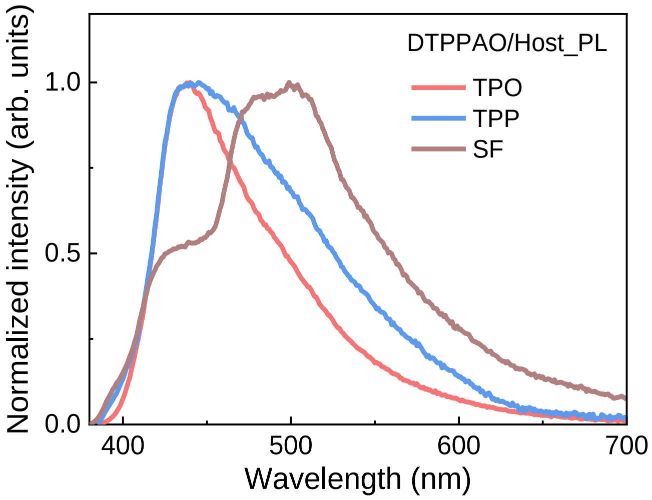

Supplementary Figure 35. Comparison of steady-state emission spectra of DTPPAO in different host matrices (TPO, TPP and SF) at ambient conditions.

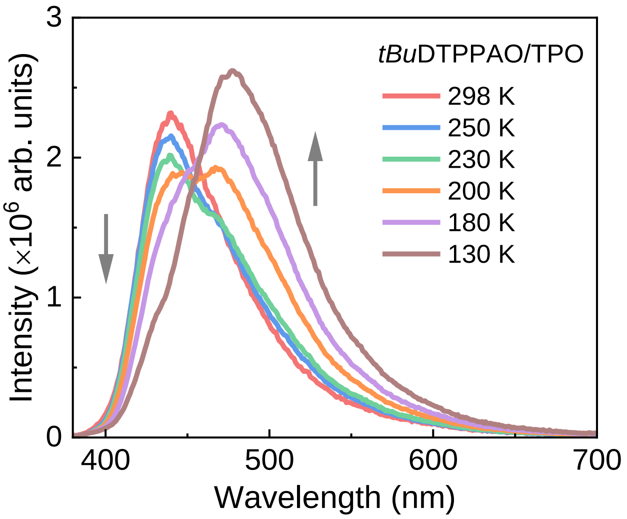

Supplementary Figure 36. Temperature-dependent emission spectra of 1 wt%*tBu*DTPPAO:TPO.

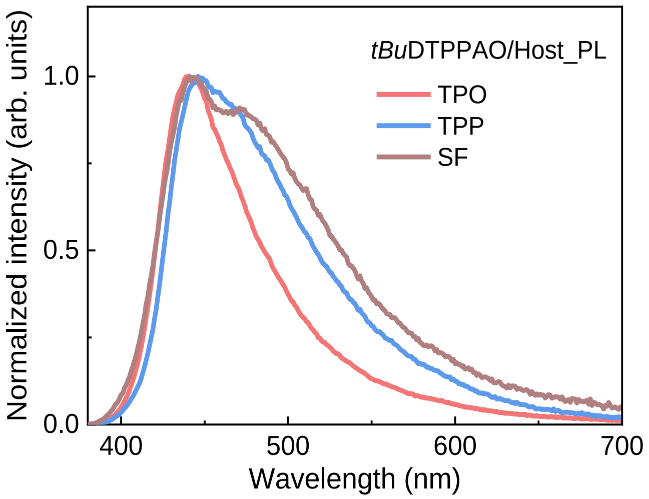

Supplementary Figure 37. Comparison of steady-state emission spectra of *tBu*DTPPAO in different host matrices (TPO, TPP and SF) at ambient conditions.

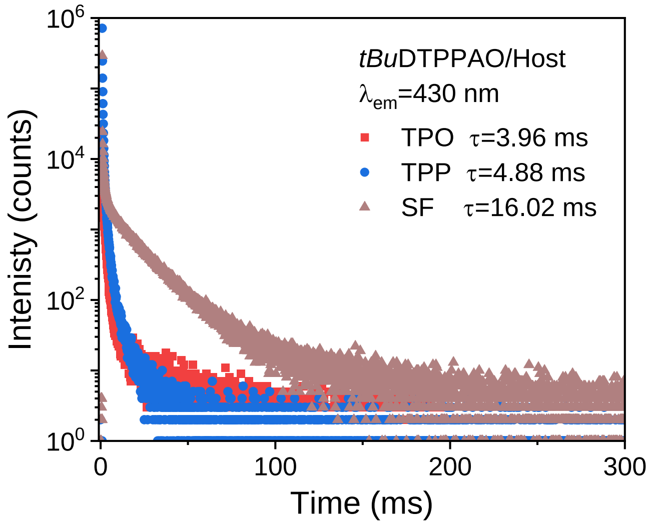

Supplementary Figure 38. Time-resolved decay curves of *tBu*DTPPAO/host recorded at the fluorescence band of 430 nm at room temperature.

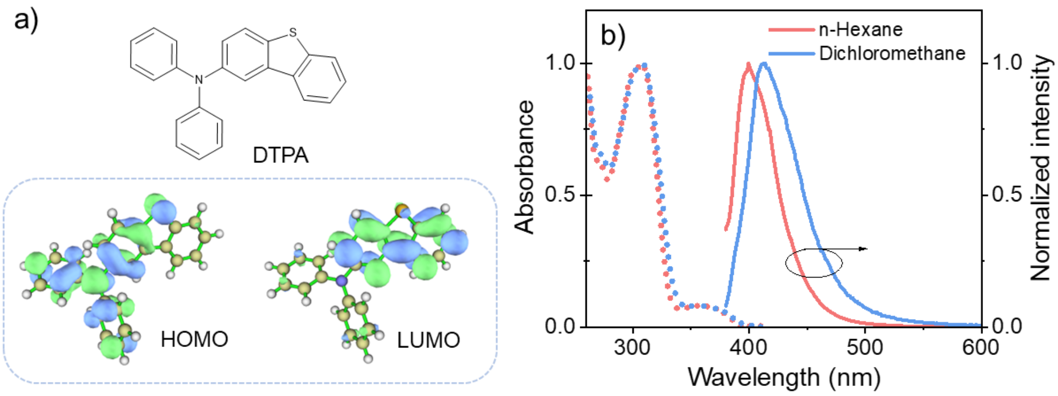

Supplementary Figure 39. a) Molecular structure of DTPA and its HOMO/LUMO distributions calculated at the B3LYP/6-311G(d) level. b) Normalized UV-vis absorption and emission spectra in n-Hexane and dichloromethane (DCM) solutions (1×10^{-5} M).

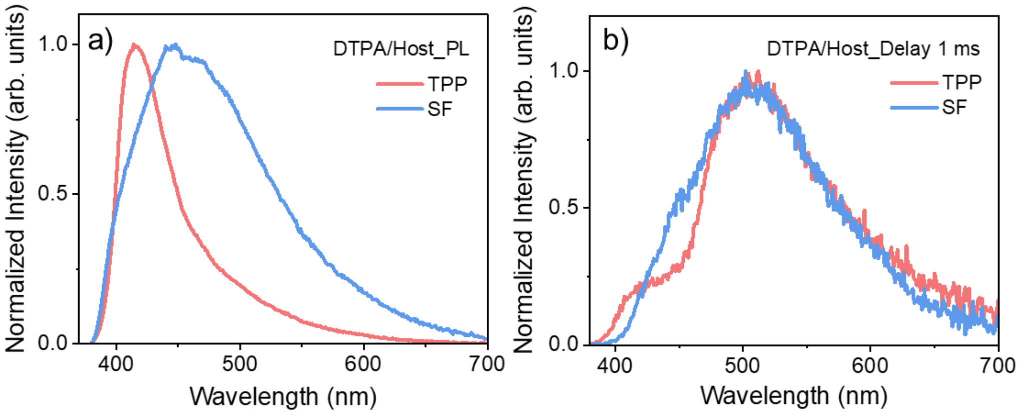

Supplementary Figure 40. a) Steady-state emission and b) delayed spectra (delay time of 1 ms) of DTPA/TPP and DTPA/SF systems (1 wt%) at ambient condition.

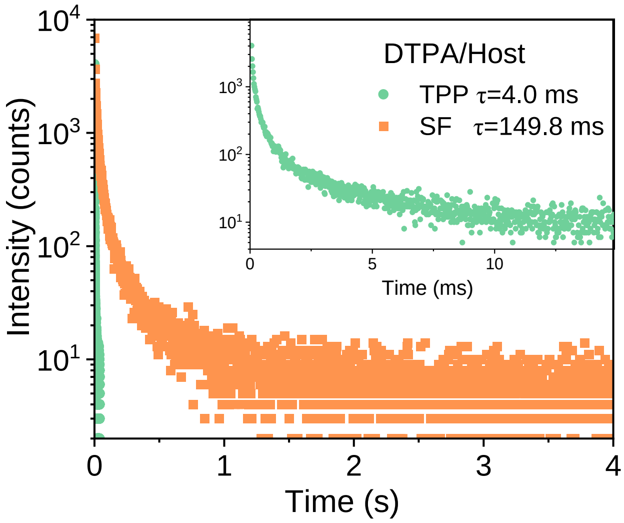

Supplementary Figure 41. Time-resolved decay curves of DTPA/TPP and DTPA/SF doping systems (1 wt%) at the phosphorescence band of 500 nm at ambient condition.

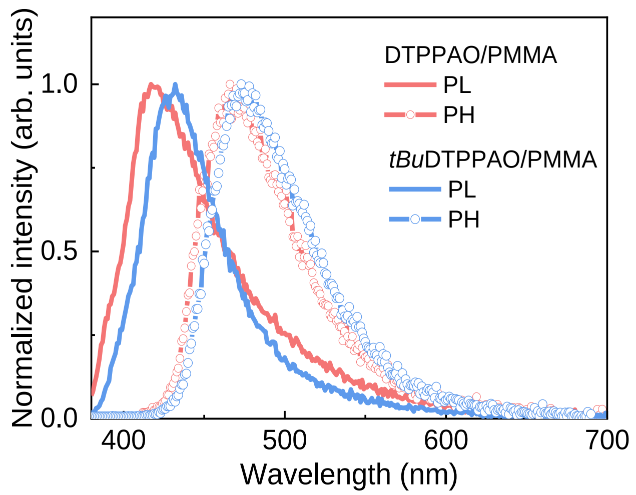

Supplementary Figure 42. Steady-state PL spectra (room temperature) and phosphorescence spectra (77 K, delay time at 8 ms) of DTPPAO and *tBu*DTPPAO doped PMMA films.

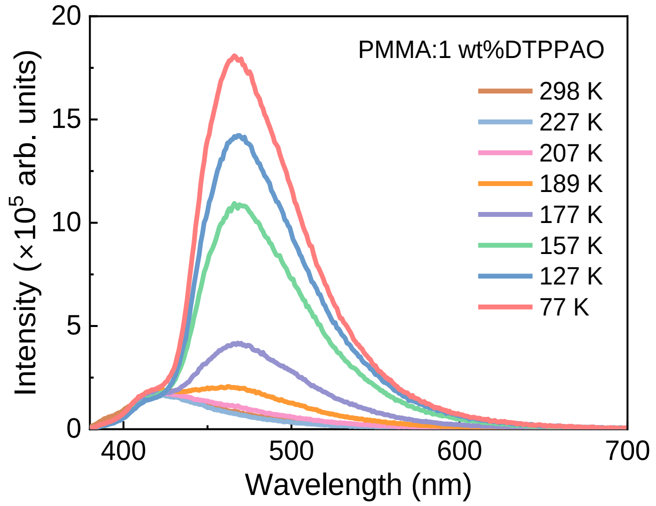

Supplementary Figure 43. Temperature-dependent emission spectra of 1 wt%DTPPAO in doped PMMA film. Excitation wavelength at 365 nm.

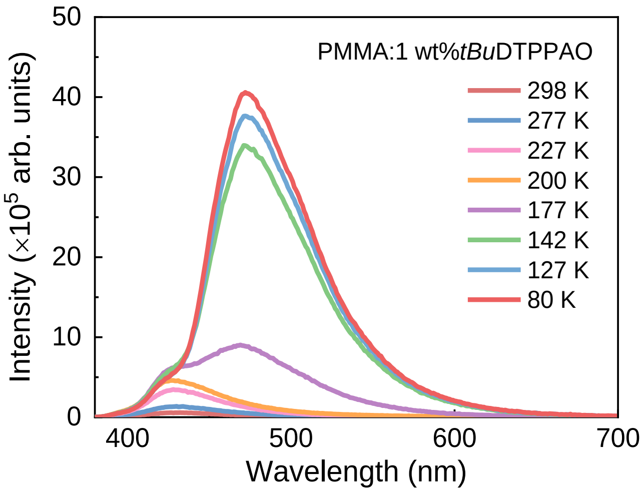

Supplementary Figure 44. Temperature-dependent emission spectra of 1 wt%*tBu*DTPPAO in doped PMMA film. Excitation wavelength at 365 nm.

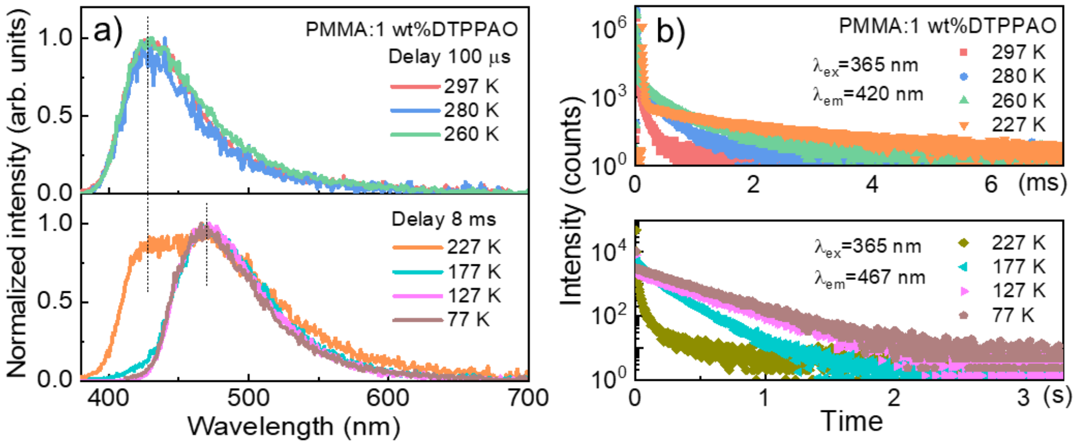

Supplementary Figure 45. PMMA:1 wt%DTPPAO film: a) Temperature-dependent delayed emission spectra of at delay time of 100 μs (top) and 8 ms (bottom). b) Temperature-dependent lifetime decay curves recorded at 420 (top) and 467 nm (bottom).

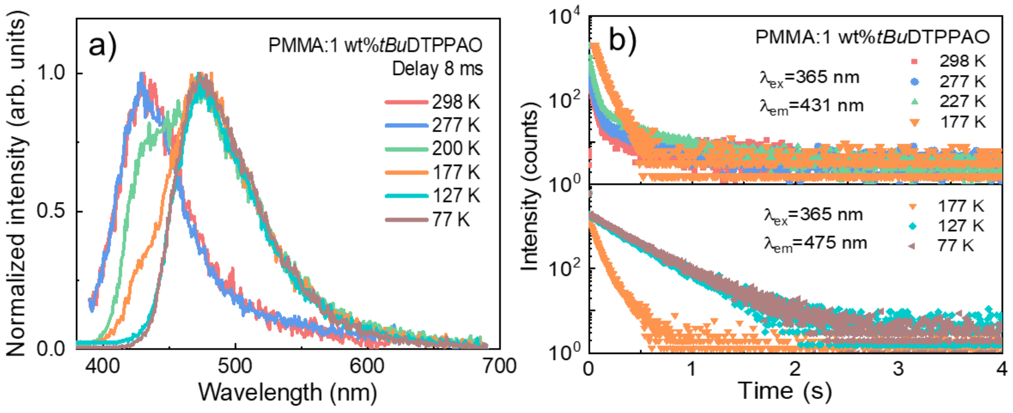

Supplementary Figure 46. PMMA:1 wt%*tBu*DTPPAO film: a) Temperature-dependent delayed emission spectra of at delay time of 8 ms. b) Temperature-dependent lifetime decay curves recorded at 431 (top) and 475 nm (bottom).

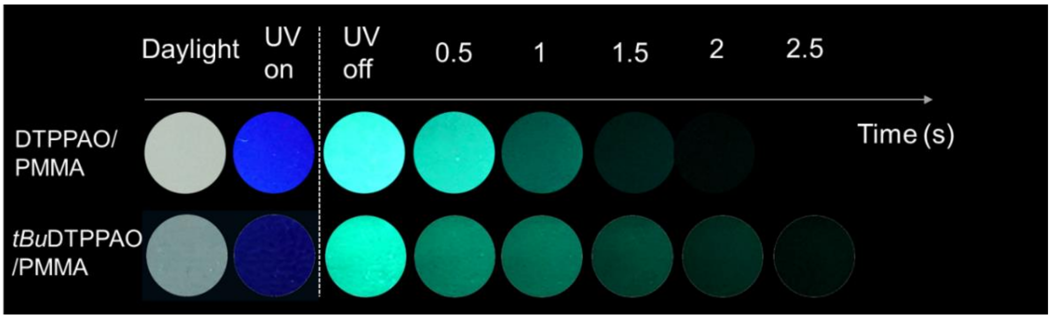

Supplementary Figure 47. Photographs of DPPAO and *tBu*DTPPAO in doped PMMA films (1 wt% doping concentration) taken under daylight and UV (365 nm) irradiation, and afterglow after the removal of UV light.

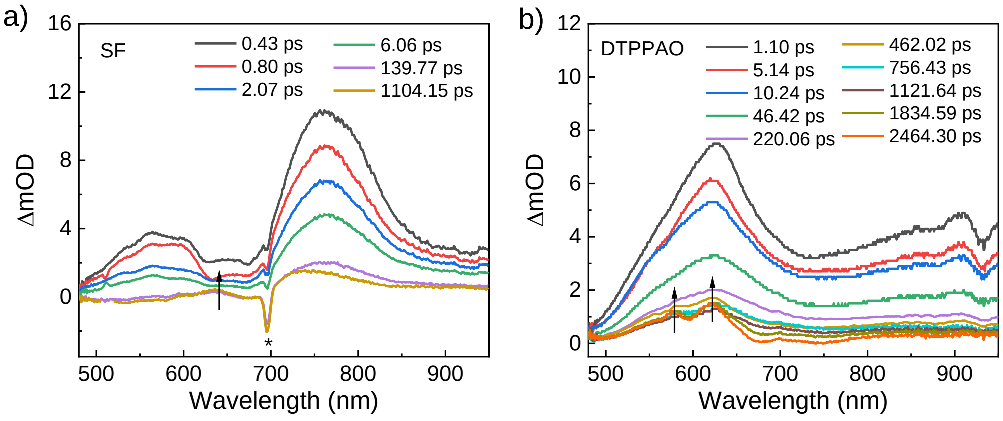

Supplementary Figure 48. Femtosecond transient absorption (fs-TA) spectra of a) SF and b) DTPPAO at different delay times upon excitation at 350 nm (The peak around 700 nm marked as * is caused by the instrument).

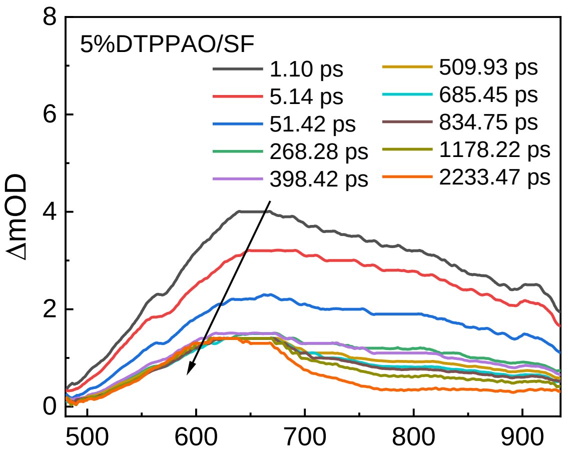

Supplementary Figure 49. Femtosecond transient absorption (fs-TA) spectra of DTPPAO/SF doping system at different delay times upon excitation at 350 nm.

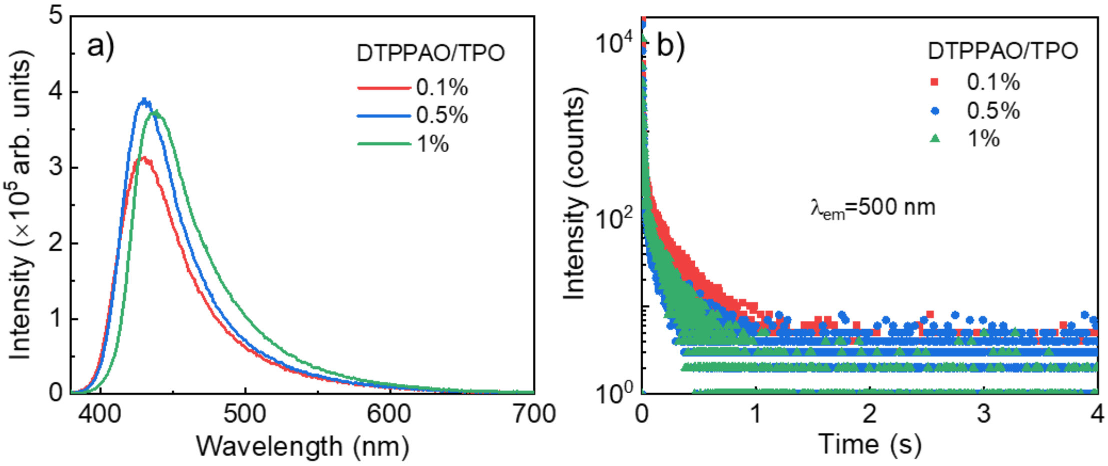

Supplementary Figure 50. a) Steady-state photoluminescence spectra and b) time-resolved decay curves recorded at 500 nm of DTPPAO/TPO doping system at different concentrations.

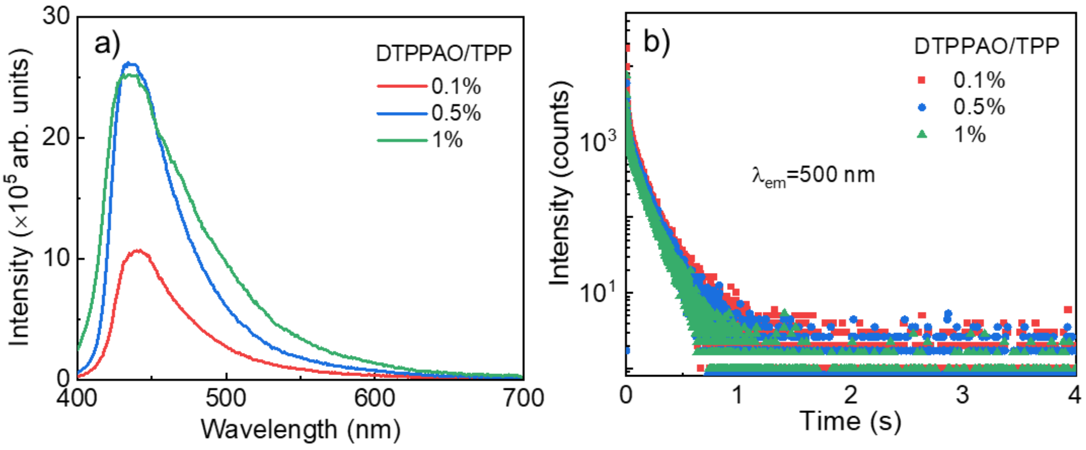

Supplementary Figure 51. a) Steady-state photoluminescence spectra and b) time-resolved decay curves recorded at 500 nm of DTPPAO/TPP doping system at different concentrations.

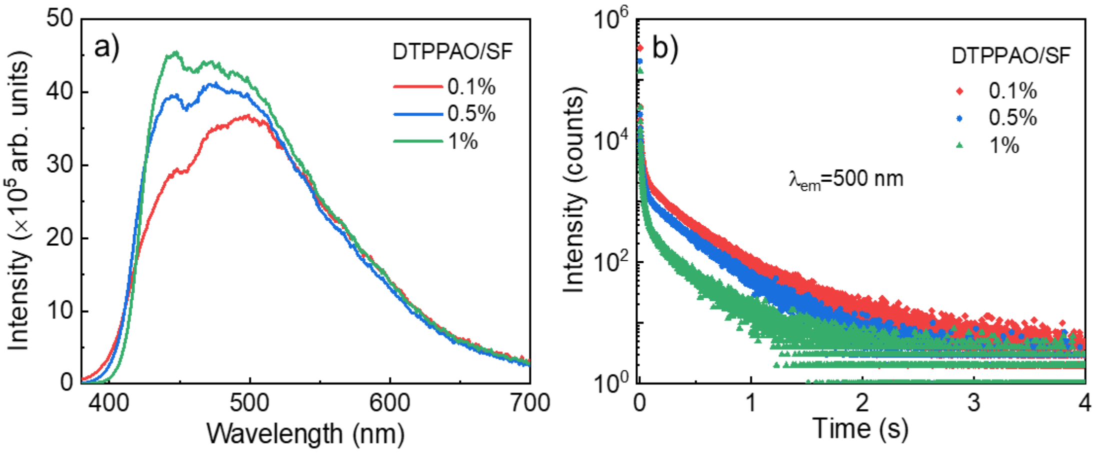

Supplementary Figure 52. a) Steady-state photoluminescence spectra and b) time-resolved decay curves recorded at 500 nm of DTPPAO/SF doping system at different concentrations.

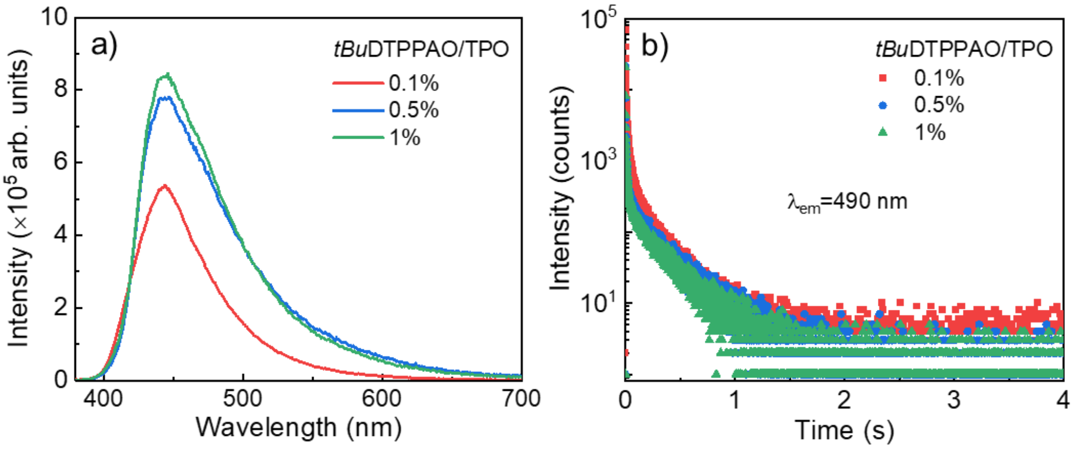

Supplementary Figure 53. a) Steady-state photoluminescence spectra and b) time-resolved decay curves recorded at 490 nm of *tBu*DTPPAO/TPO doping system at different concentrations.

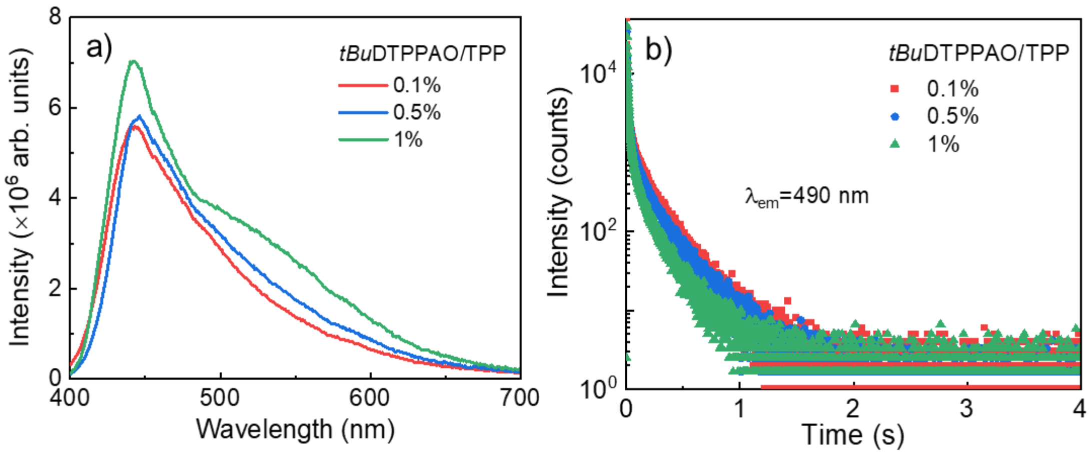

Supplementary Figure 54. a) Steady-state photoluminescence spectra and b) time-resolved decay curves recorded at 490 nm of *tBu*DTPPAO/TPP doping system at different concentrations.

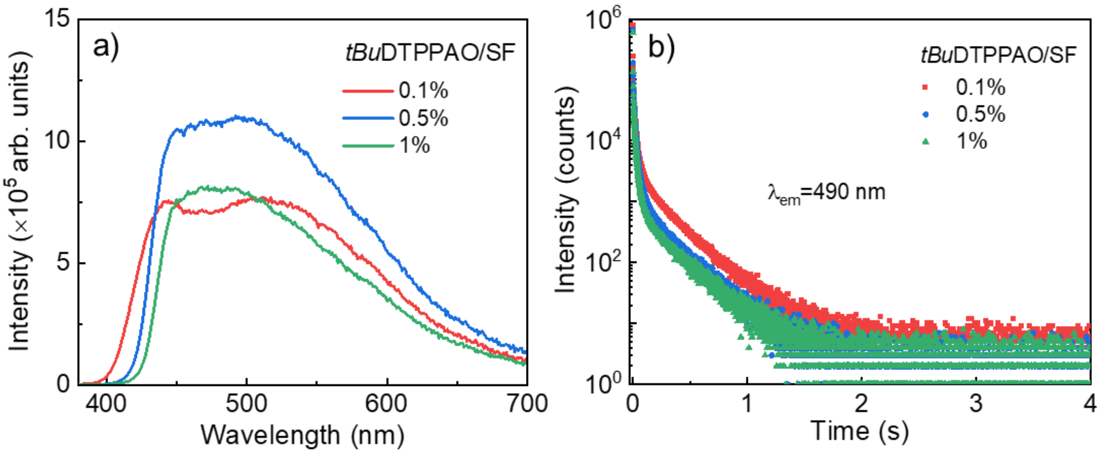

Supplementary Figure 55. a) Steady-state photoluminescence spectra and b) time-resolved decay curves recorded at 490 nm of *tBu*DTPPAO/SF doping system at different concentrations.

1.4 Theoretical Calculations

The frontier molecular orbital (FMO) distributions and excitation characteristics of guest (DTPPAO and *tBu*DTPPAO) and host matrices (TPO, TPP and SF) were investigated by density functional theoretical (DFT) and time-dependent DFT (TD-DFT) calculations based on optimized molecular structures in gas phase at the B3LYP/6-311G(d) level.[1-2] All computations were performed using the Gaussian 09 package.[3] The HOMO and LUMO distributions of DTPPAO and *tBu*DTPPAO are effectively decoupled on the D and A units, respectively, which reveals a CT character of the ground state. The HOMO energy levels of DTPPAO and *tBu*DTPPAO are basically in accord with their donor units, -5.38 eV for DPA and -5.21 eV for *tBu*DPA, revealing a stronger electron-donating effect of tert-butyl groups. To get a better insight into the nature of the excited states, the natural transition orbitals (NTOs) and spin-orbital coupling (SOC) process were also performed according to the geometry optimization in the singlet and triplet excited states.[1]

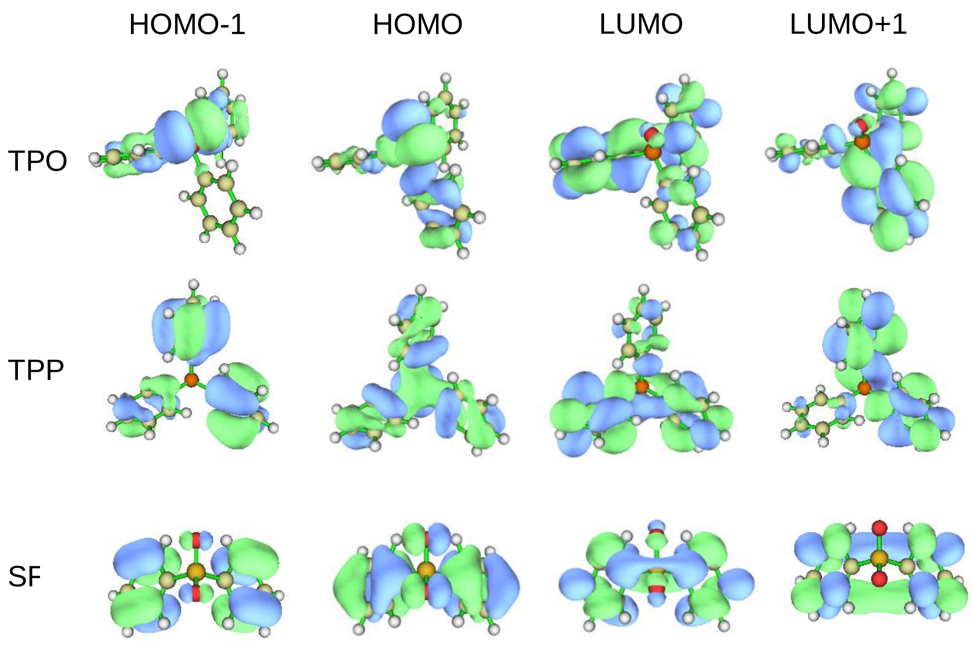

Supplementary Figure 56. Frontier molecular orbitals based on optimized geometries of TPO, TPP and SF with B3LYP/6-311G(d) functionals.

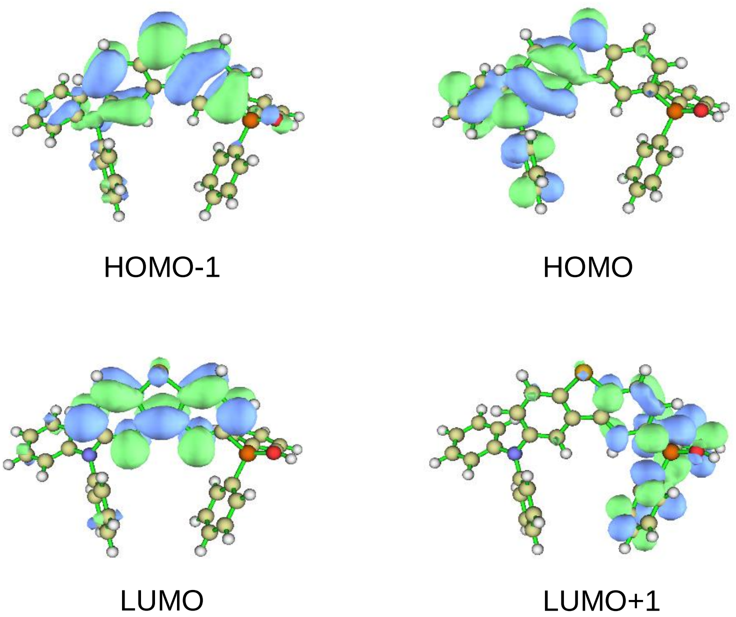

Supplementary Figure 57. Frontier molecular orbitals based on optimized geometries of DTPPAO with B3LYP/6-311G(d) functionals.

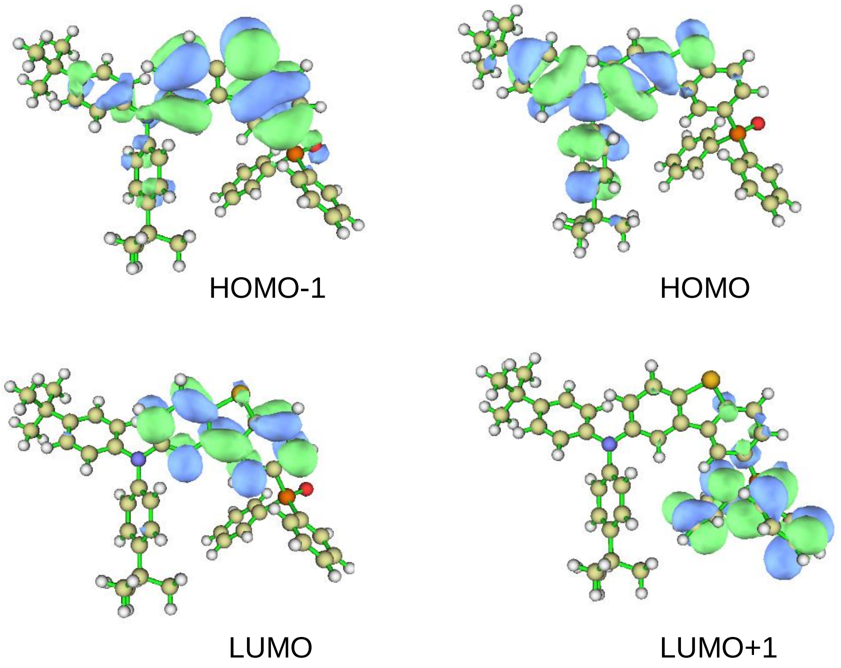

Supplementary Figure 58. Frontier molecular orbitals based on optimized geometries of *tBu*DTPPAO with B3LYP/6-311G(d) functionals.

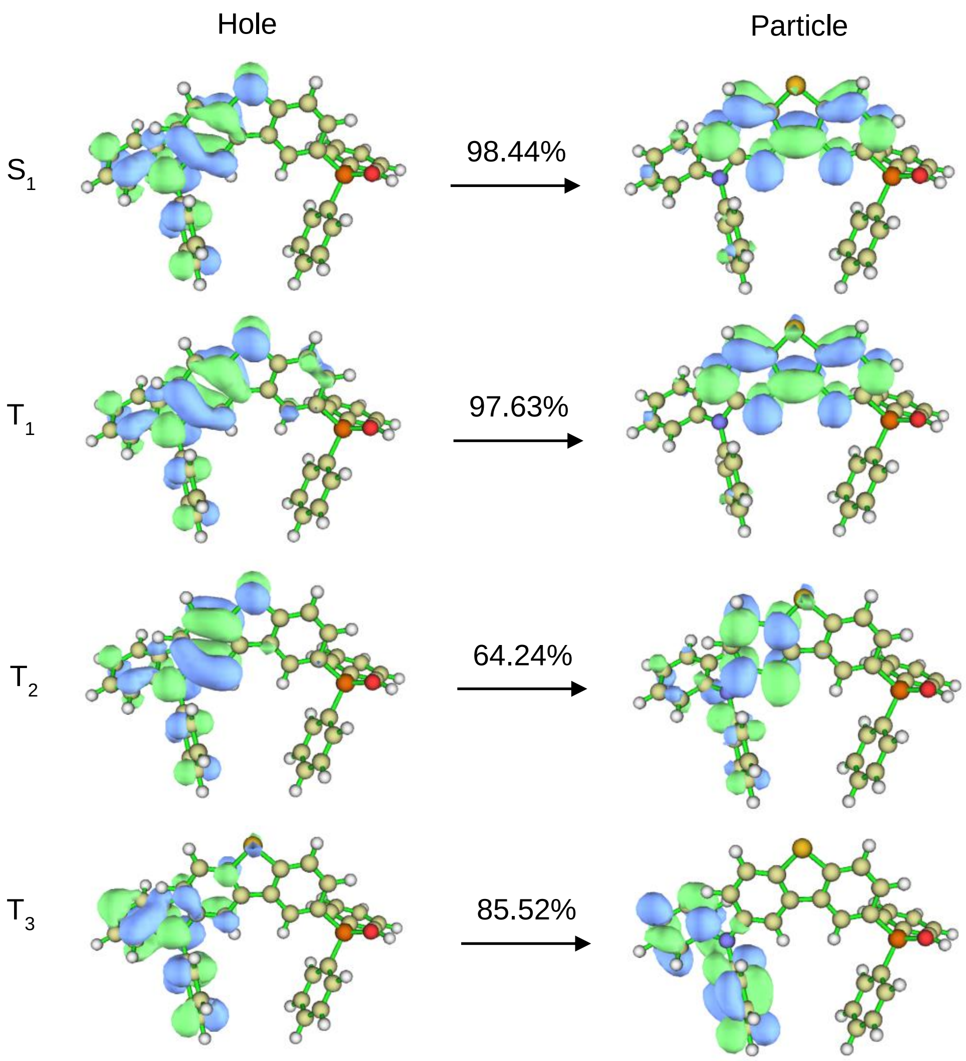

Supplementary Figure 59. Natural transition orbitals (NTO) describing the excitation characters of the S_1, T_1, T_2 and T_3 states in DTPPAO; the weights of the hole-electron contributions to the excitations are included.

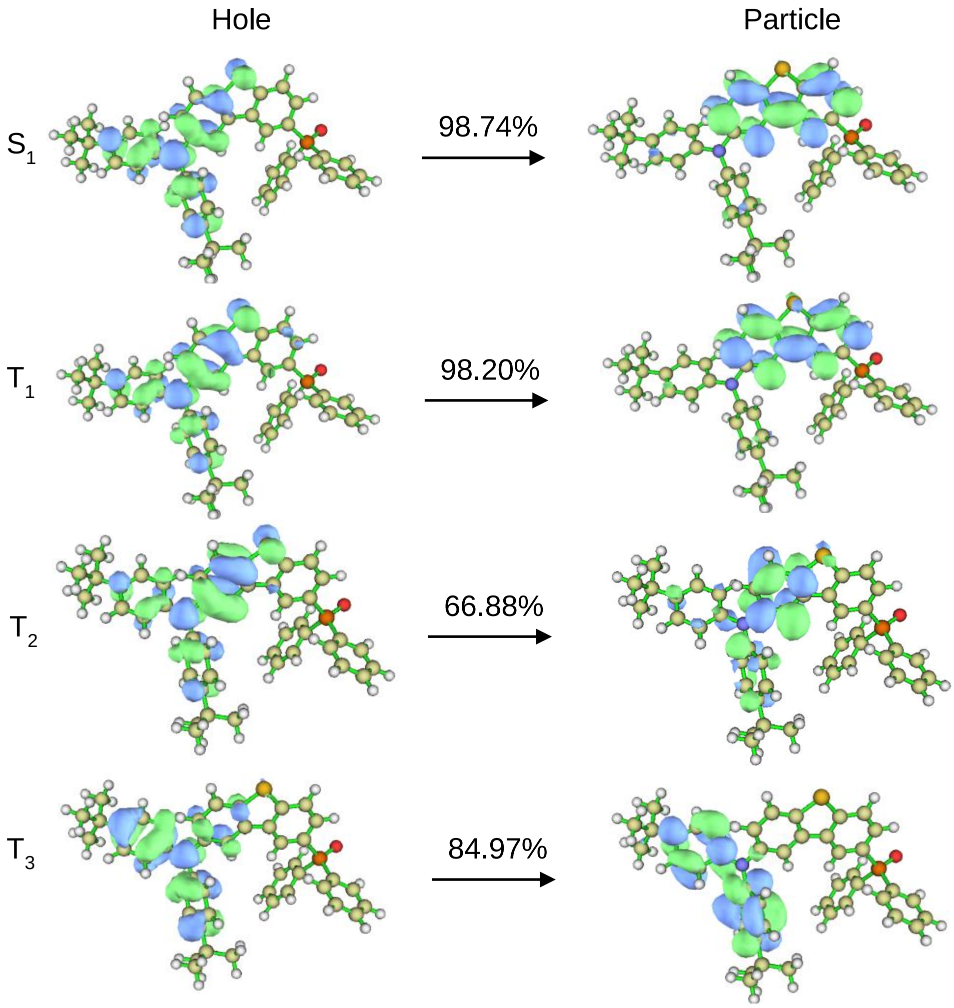

Supplementary Figure 60. Natural transition orbitals (NTO) describing the excitation characters of the S_1, T_1, T_2 and T_3 states in *tBu*DTPPAO; the weights of the hole-electron contributions to the excitations are included.

1.5 3D printing structures

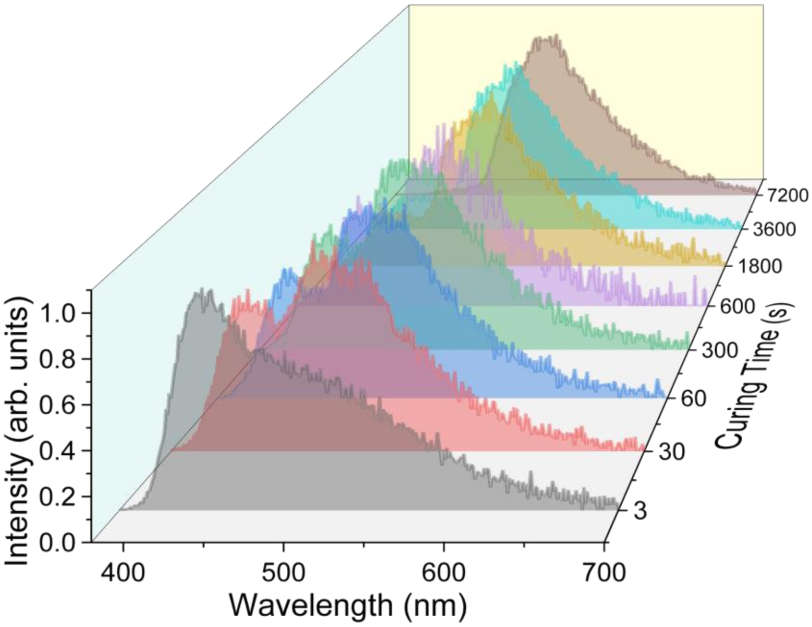

Supplementary Figure 61. Normalized delayed emission spectra of 0.1 wt% DTPPAO/HEA-AA film (1 mm) at different photocuring time (3, 30, 60, 300, 600, 1800, 3600, 7200 s) collected at a delay time of 8 ms.

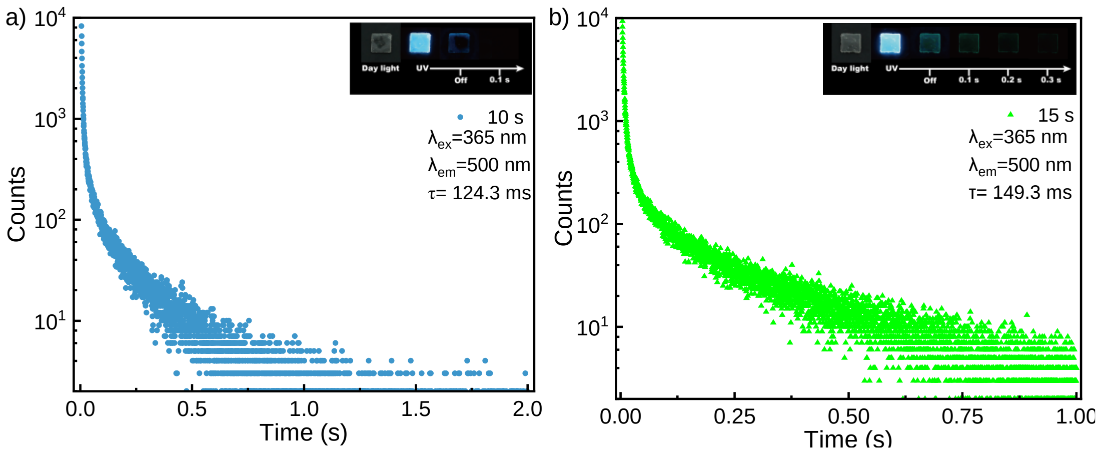

Supplementary Figure 62. Lifetime decay curves and digital photos of 0.1 wt% DTPPAO/HEA-AA film at the photocuring times of a) 10 s and b) 15 s.

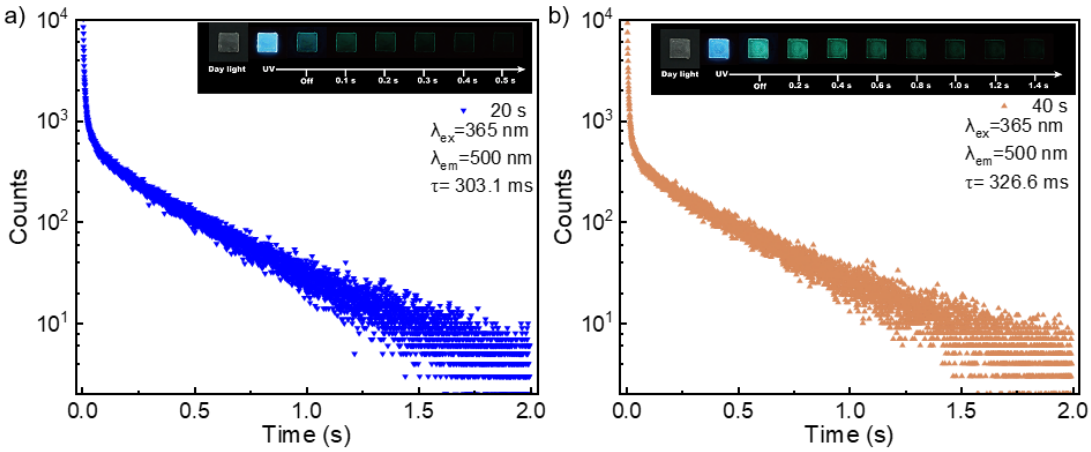

Supplementary Figure 63. Lifetime decay curves and digital photos of 0.1 wt% DTPPAO/HEA-AA film at the photocuring times of a) 20 s and b) 40 s.

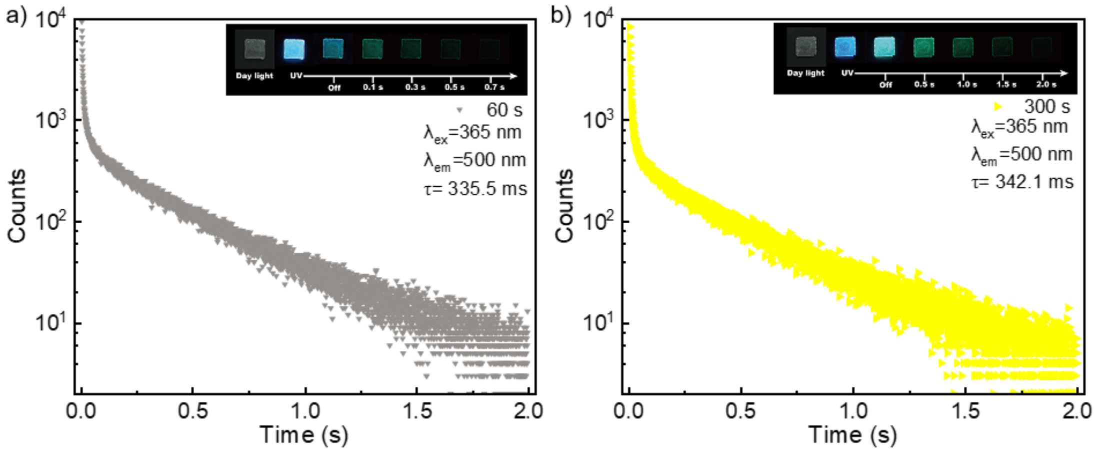

Supplementary Figure 64. Lifetime decay curves and digital photos of 0.1 wt% DTPPAO/HEA-AA film at the photocuring times of a) 60 s and b) 300 s.

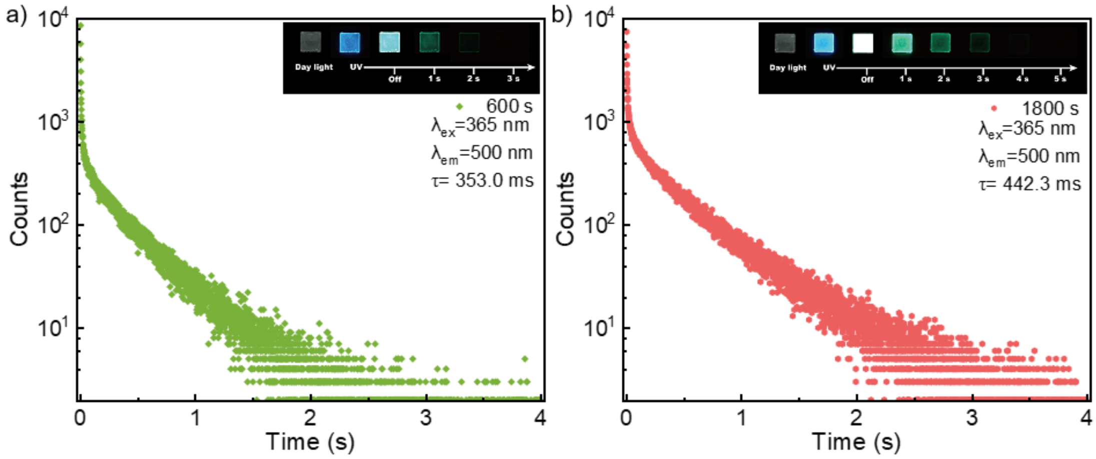

Supplementary Figure 65. Lifetime decay curves and digital photos of 0.1 wt% DTPPAO/HEA-AA film at the photocuring times of a) 600 s and b) 1800 s.

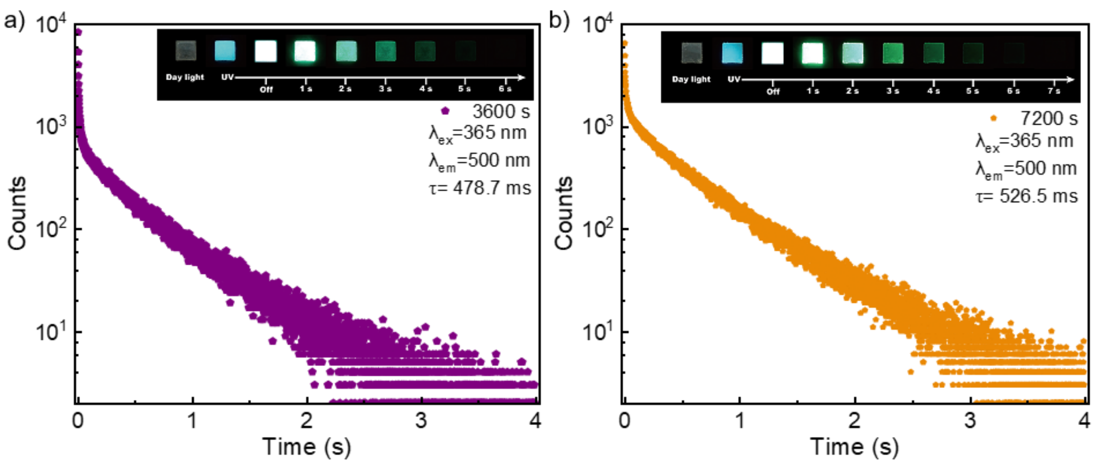

Supplementary Figure 66. Lifetime decay curves and digital photos of 0.1 wt% DTPPAO/HEA-AA film at the photocuring times of a) 3600 s and b) 7200 s.

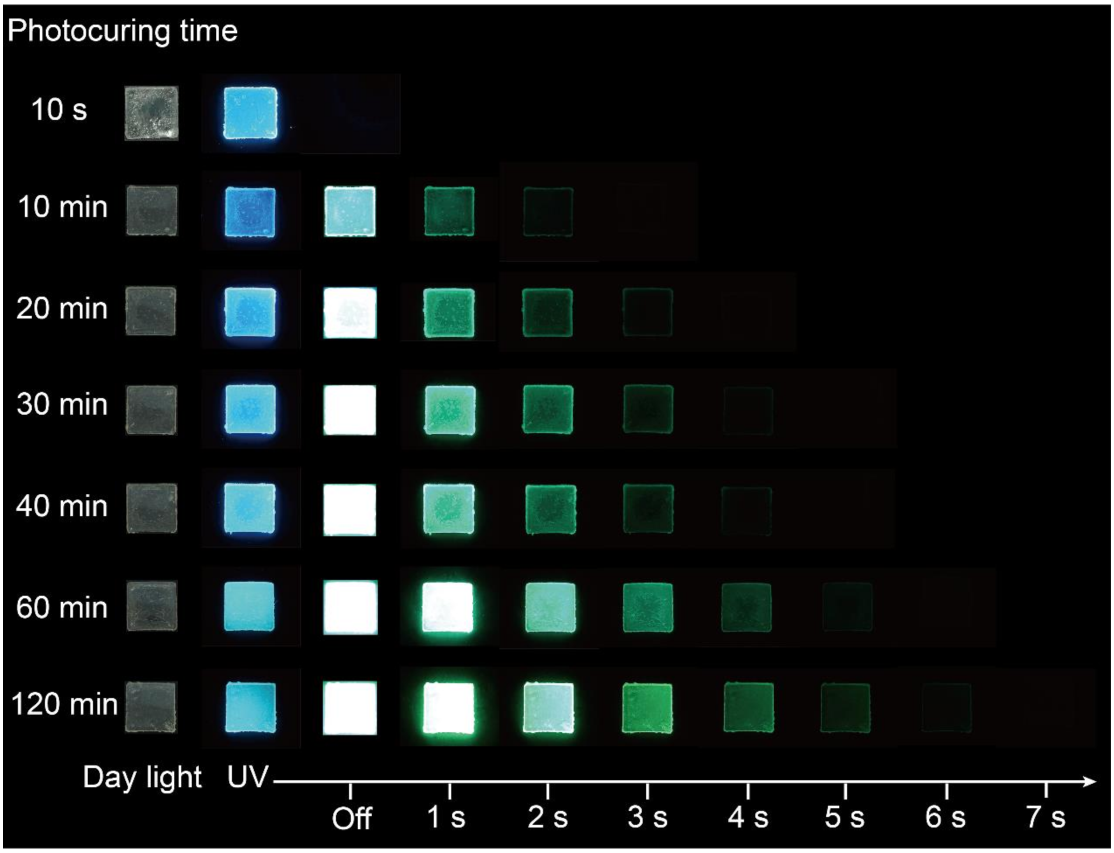

Supplementary Figure 67. Photographs of DTPPAO/HEA-AA film with different photocuring times taken under a 365 nm UV lamp on and off.

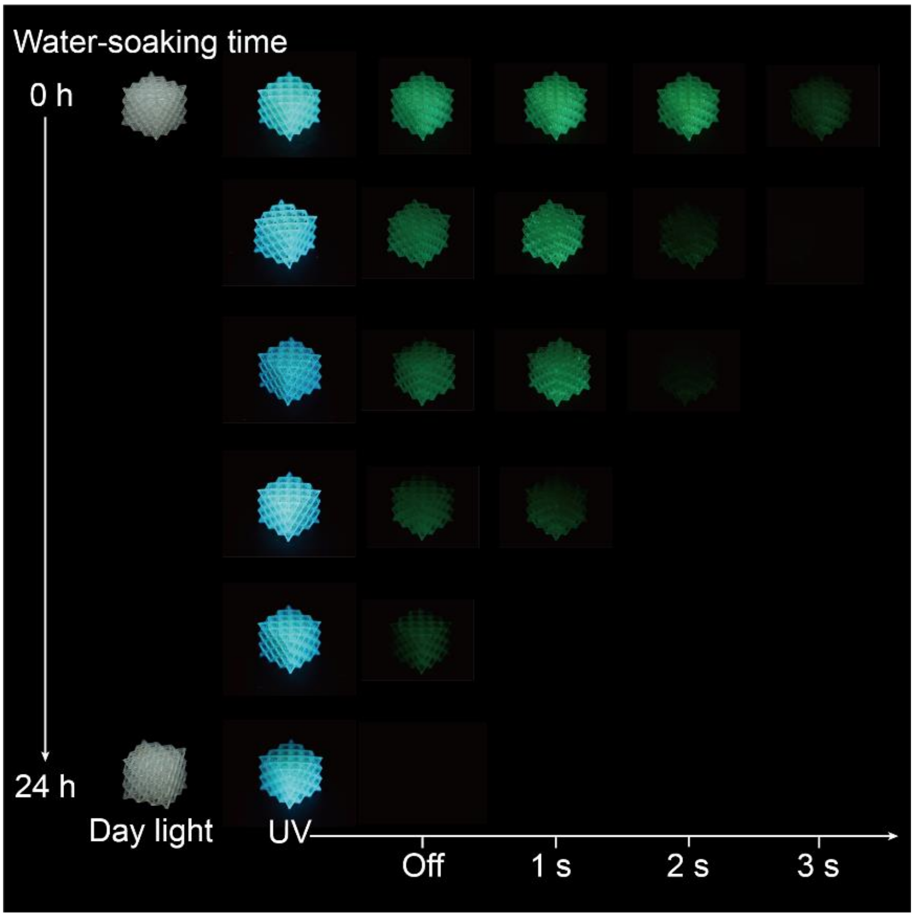

Supplementary Figure 68. Digital photographs of 3D printing structures placed in a humid environment (supersaturated NaBr solution, 58%RH) with different water-soaking time taken under a 365 nm UV lamp on and off.

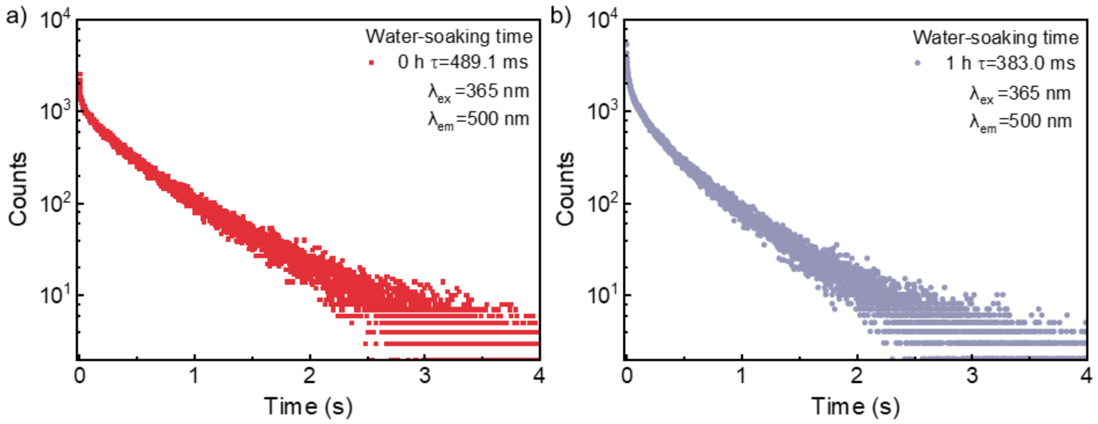

Supplementary Figure 69. Lifetime decay curves of DTPPAO/HEA-AA film at a) initial time and b) water-soaking time of 1h.

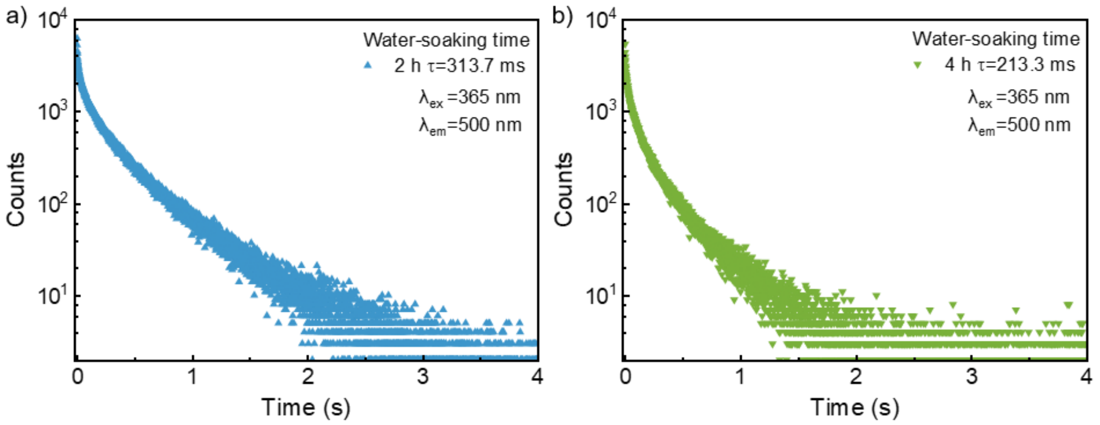

Supplementary Figure 70. Lifetime decay curves of DTPPAO/HEA-AA film with the water-soaking times of a) 2 h and b) 4 h.

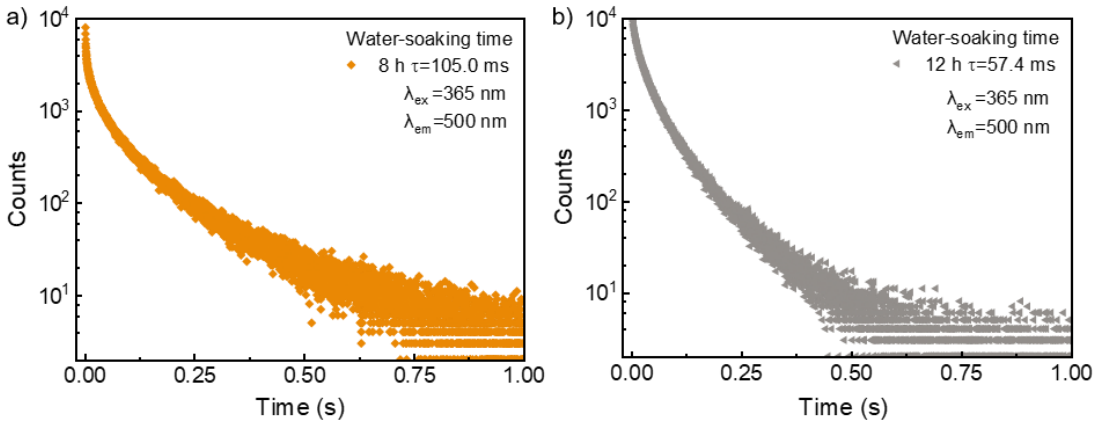

Supplementary Figure 71. Lifetime decay curves of DTPPAO/HEA-AA film with the water-soaking times of a) 8 h and b) 12 h.

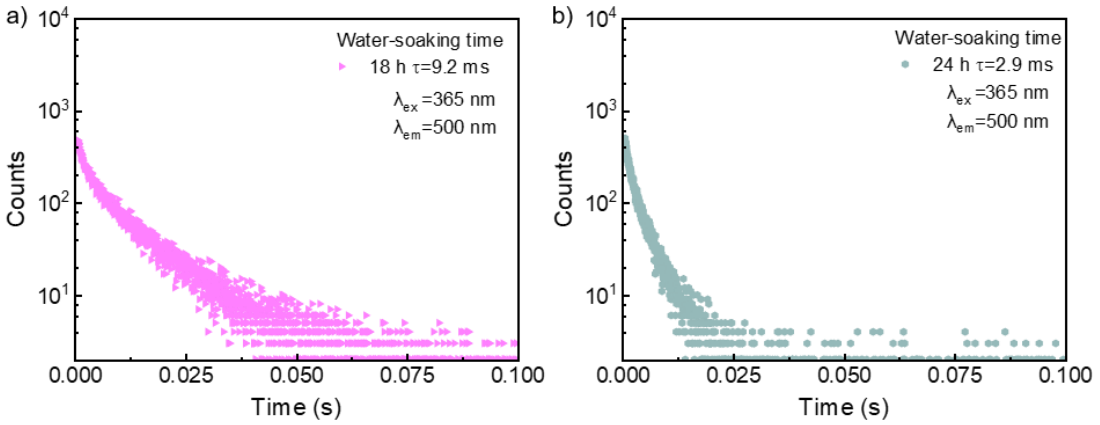

Supplementary Figure 72. Lifetime decay curves of DTPPAO/HEA-AA film with the water-soaking time of a) 18 h and b) 24 h.

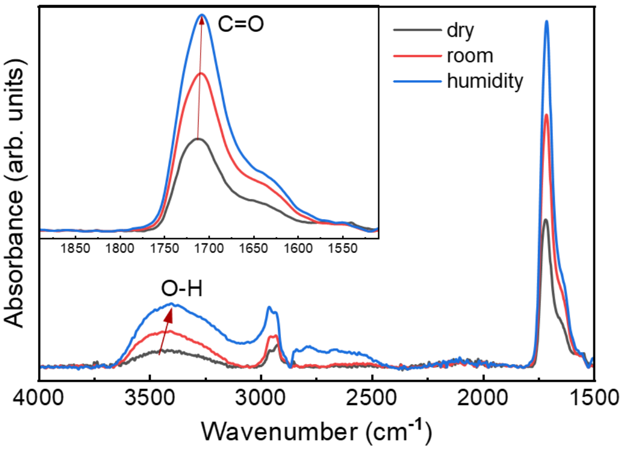

Supplementary Figure 73. Fourier transform infrared (FTIR) spectra in DTPPAO/HEA-AA film under the heating and water fuming stimuli (dry: drying treatment at oven; room: at ambient condition for 24 h; humidity: at 58%RH moisture supersaturated NaBr solution for 24 h).

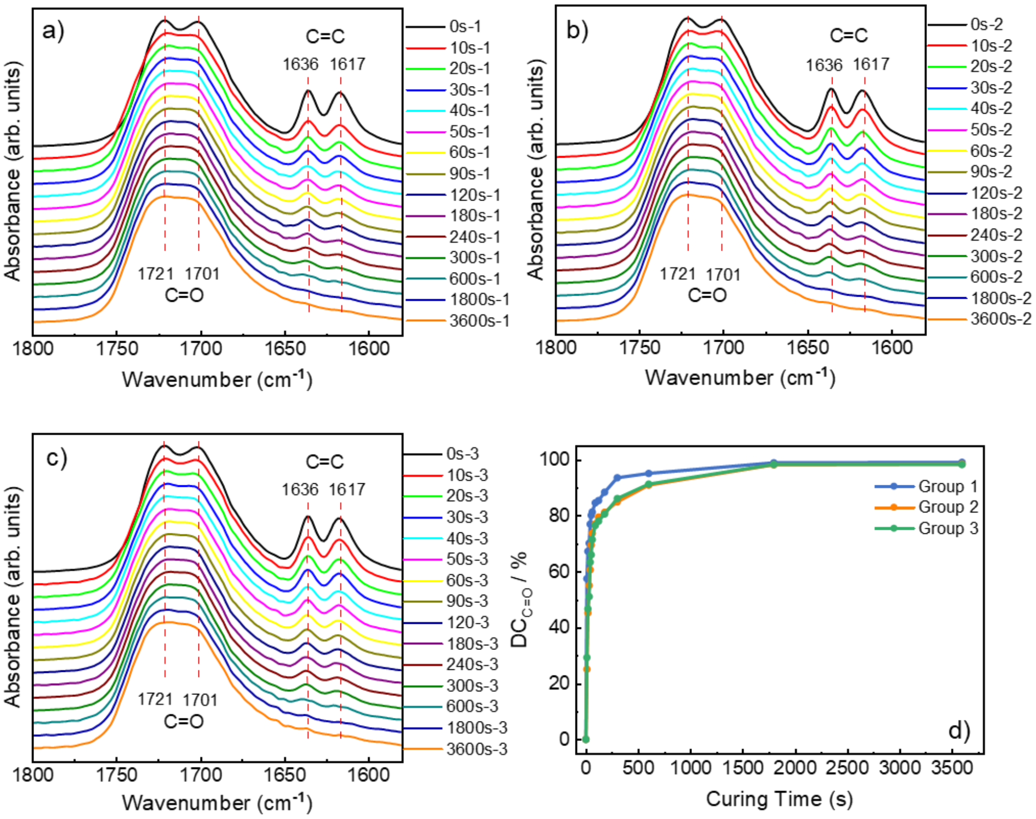

Supplementary Figure 74. The Fourier transform infrared (FTIR) spectra at the range of 1580-1800 cm^{-1}. FTIR spectra of DTPPAO/HEA-AA at different curing times (0, 10, 20, 30, 40, 50, 60, 90, 120, 180, 240, 300, 600, 1800, 3600 s) of a) Group 1, b) Group 2 and c) Group 3. d) C=C bond conversion rate ($DC_{C=O}$) of the three groups at different photocuring time.

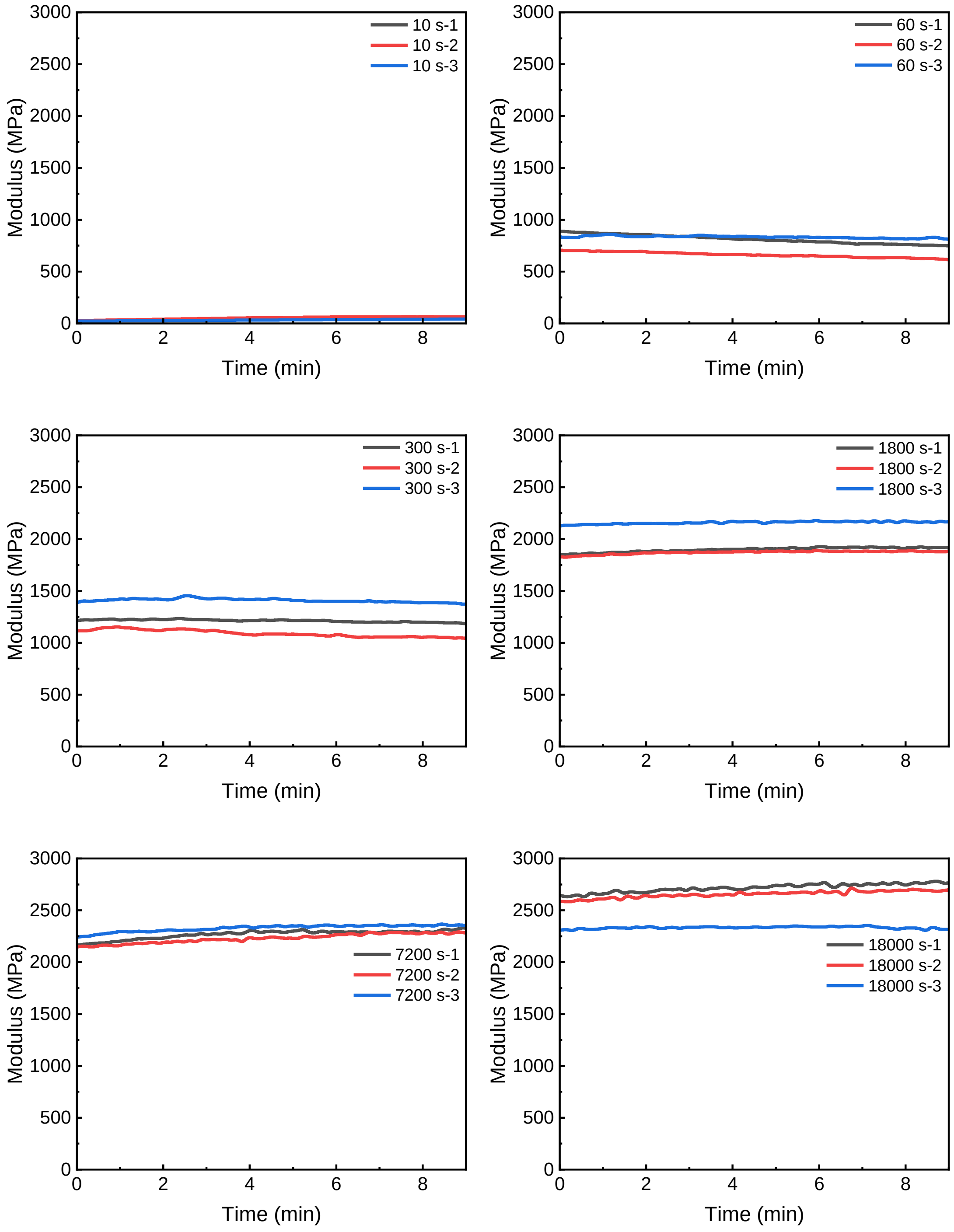

Supplementary Figure 75. The Storage Modulus of DTPPAO/HEA-AA film at different photocuring times (10, 60, 300,1800, 7200, 18000 s).

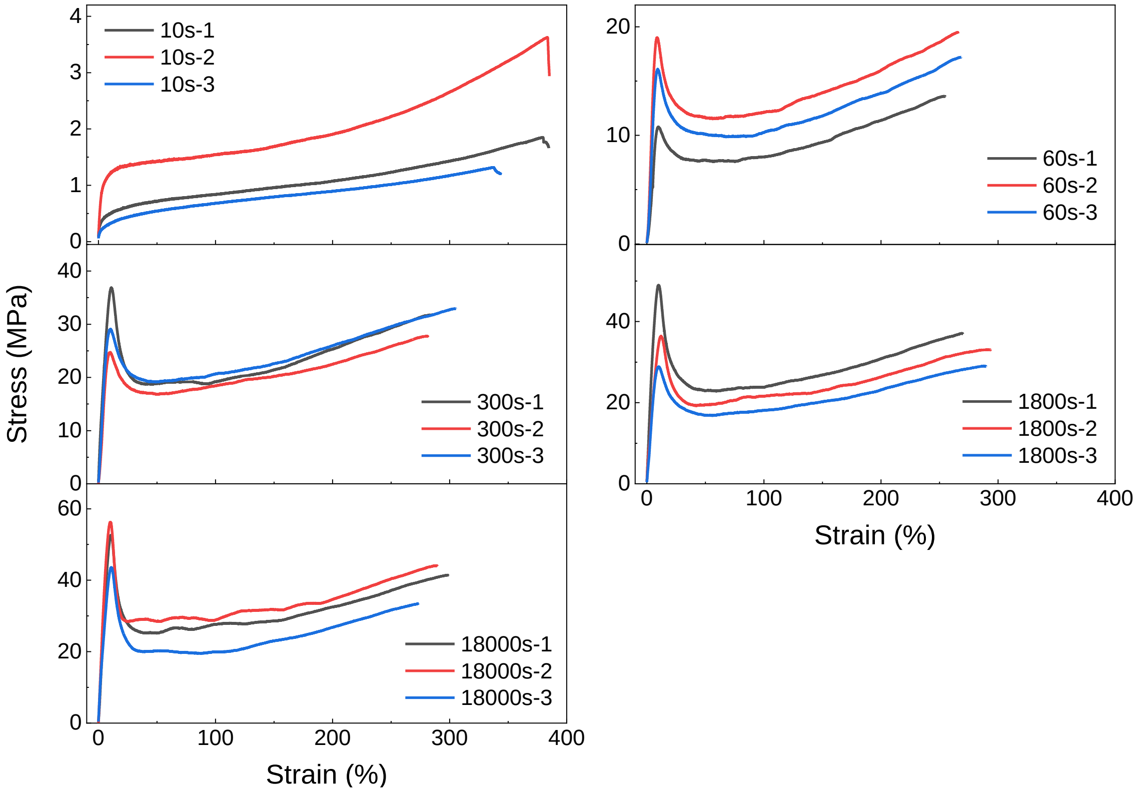

Supplementary Figure 76. Mechanical tensile curves of DTPPAO/HEA-AA at different photocuring time (10, 60, 300, 1800, 18000 s)

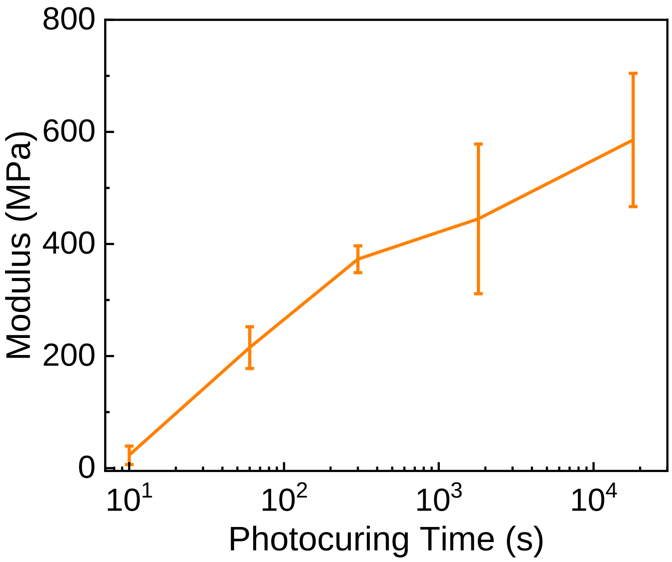

Supplementary Figure 77. Young's modulus of DTPPAO/HEA-AA at different photocuring times (10, 60, 300, 1800, 18000 s)

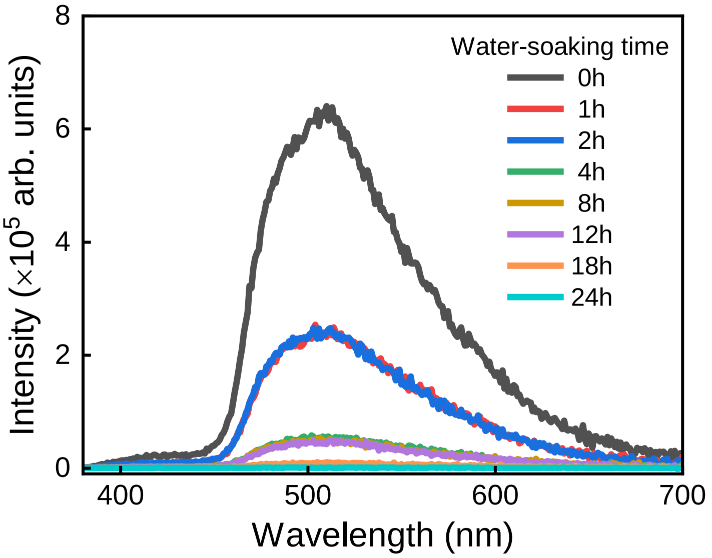

Supplementary Figure 78. Delayed emission spectra of DTPPAO/HEA-AA film left in humid environment (58RH%) for different times (0, 1, 2, 4, 8, 12, 18, 24 h).

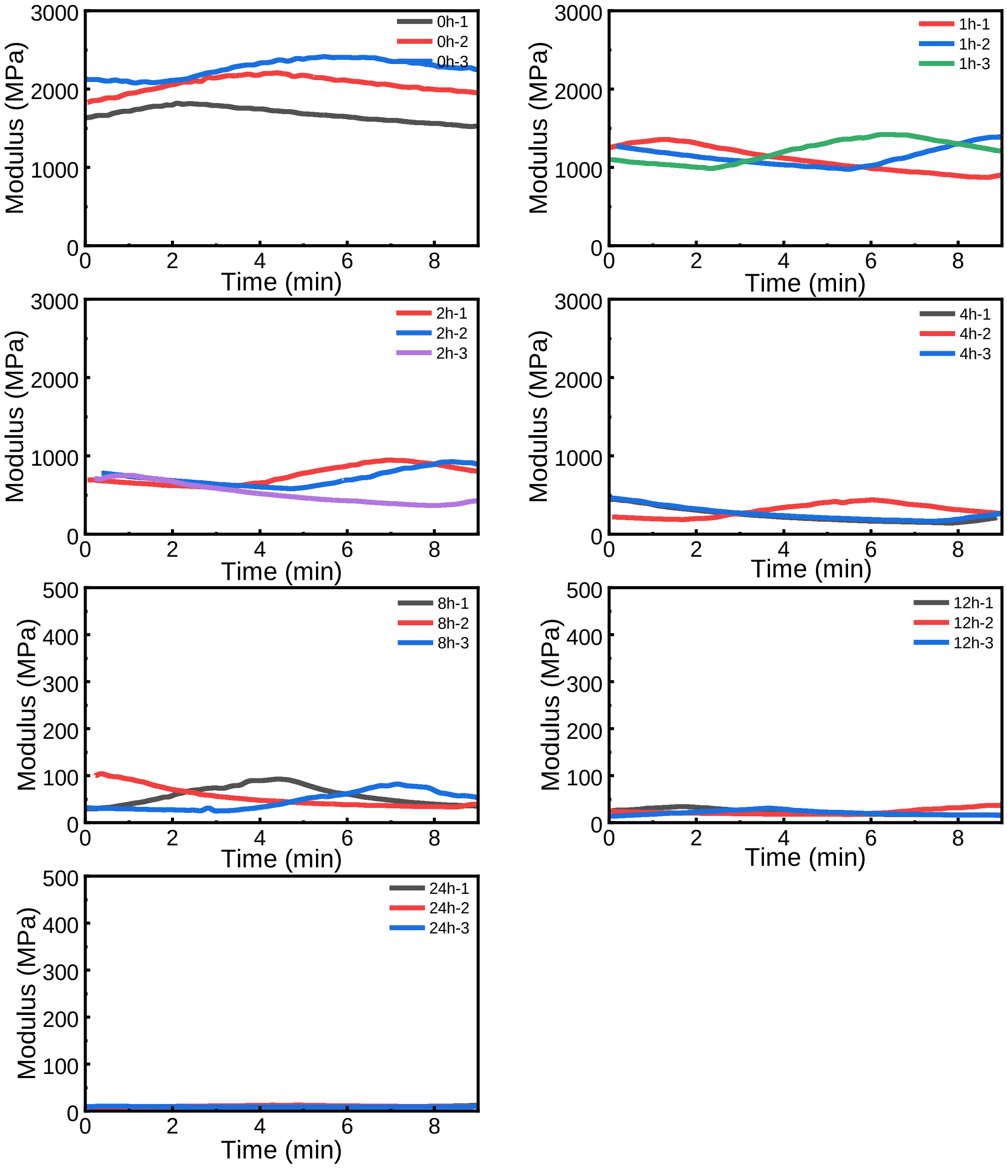

Supplementary Figure 79. Storage Modulus of DTPPAO/HEA-AA (photocuring time: 5 h) left in humid environment (58%RH) for different times (0, 1, 2, 4, 8, 12, 24 h).

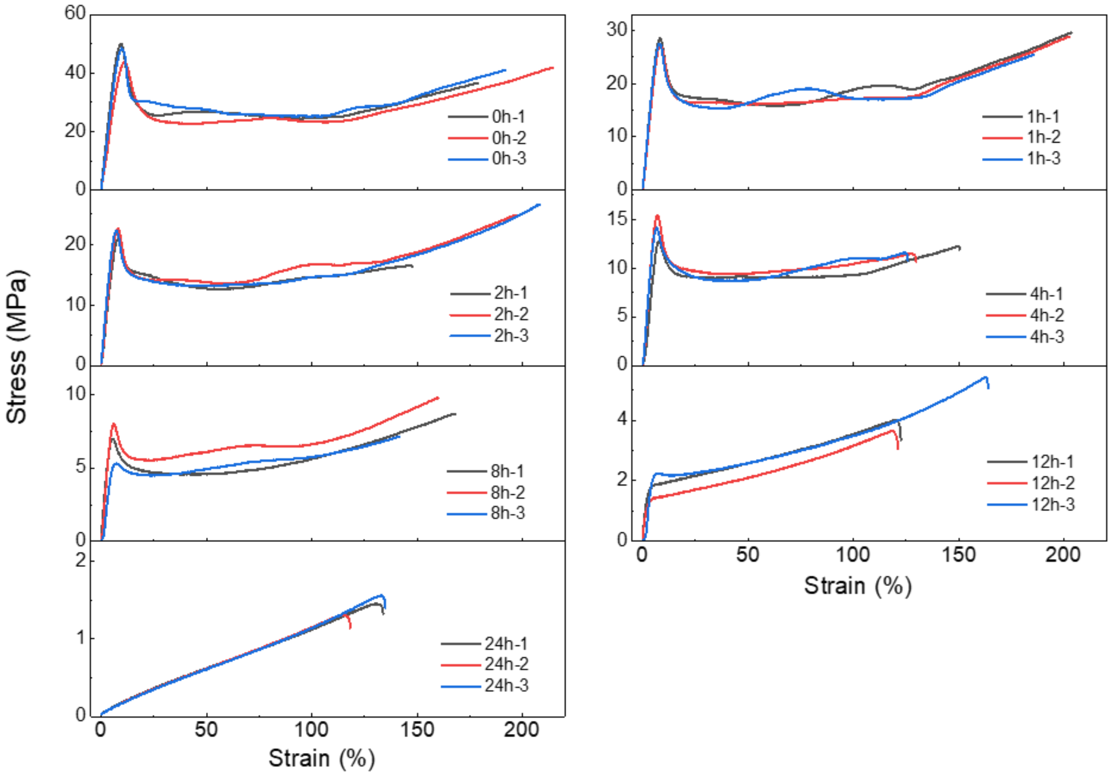

Supplementary Figure 80. Mechanical tensile curves of DTPPAO/HEA-AA (photocuring time: 5 h) left in humid environment (58%RH) for different times (0, 1, 2, 4, 8, 12, 24 h).

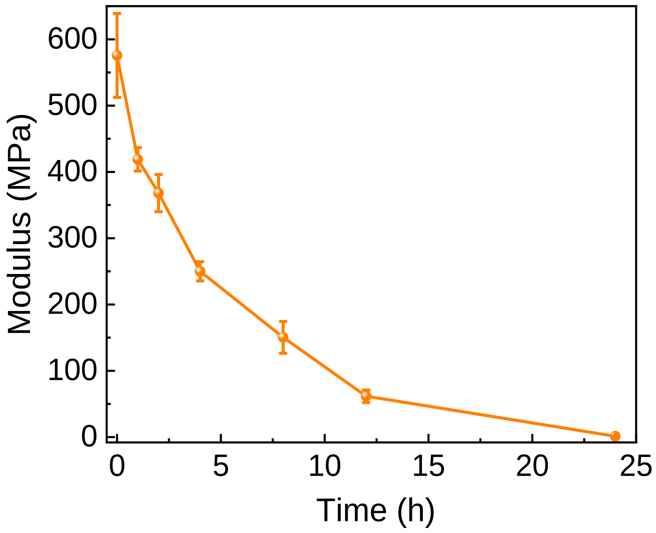

Supplementary Figure 81. Young's Modulus of DTPPAO/HEA-AA (photocuring time: 5 h) left in humid environment (58%RH) for different times (0, 1, 2, 4, 8, 12, 24 h).

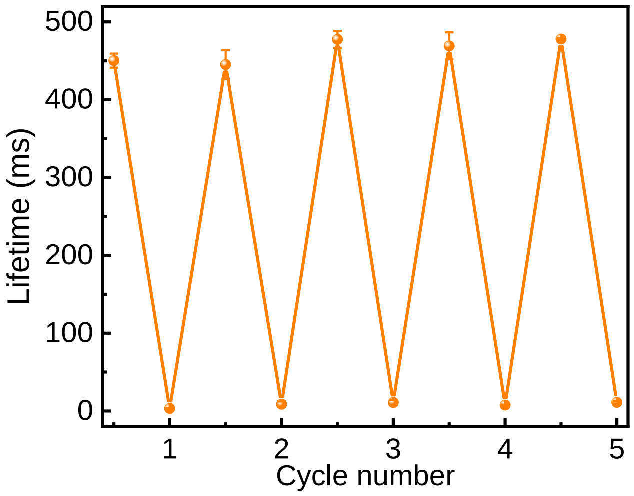

Supplementary Figure 82. The phosphorescent lifetimes of DTPPAO/HEA-AA after repeated cycles water-absorbing water and drying processes.

S2. Supplementary References

1. Lu, T. & Chen, F. Multiwfn: A multifunctional wavefunction analyzer. *J Comput. Chem.* **33**, 580–592 (2012).

2. Gao, F. *et al.* High-efficiency blue thermally activated delayed fluorescence from donor-acceptor-donor systems: Via the through-space conjugation effect. *Chem. Sci.* **10**, 5556–5567 (2019).

3. Gaussian 16, Revision C.01, M. J. Frisch, G. W. Trucks, H. B. Schlegel, G. E. Scuseria, M. A. Robb, J. R. Cheeseman, G. Scalmani, V. Barone, G. A. Petersson, H. Nakatsuji, X. Li, M. Caricato, A. Marenich, J. Bloino, B. G. Janesko, R. Gomperts, B. Mennucci, H. P. Hratchian, J. V. Ortiz, A. F. Izmaylov, J. L. Sonnenberg, D. Williams-Young, F. Ding, F. Lipparini, F. Egidi, J. Goings, B. Peng, A. Petrone, T. Henderson, D. Ranasinghe, V. G. Zakrzewski, J. Gao, N. Rega, G. Zheng, W. Liang, M. Hada, M. Ehara, K. Toyota, R. Fukuda, J. Hasegawa, M. Ishida, T. Nakajima, Y. Honda, O. Kitao, H. Nakai, T. Vreven, K. Throssell, J. A. Montgomery, Jr., J. E. Peralta, F. Ogliaro, M. Bearpark, J. J. Heyd, E. Brothers, K. N. Kudin, V. N. Staroverov, T. Keith, R. Kobayashi, J. Normand, K. Raghavachari, A. Rendell, J. C. Burant, S. S. Iyengar, J. Tomasi, M. Cossi, J. M. Millam, M. Klene, C. Adamo, R. Cammi, J. W. Ochterski, R. L. Martin, K. Morokuma, O. Farkas, J. B. Foresman, and D. J. Fox, Gaussian, Inc., Wallingford CT, 2016.
