# Supplementary material for: Tunable afterglow for mechanical self-monitoring 3D printing structures
Source: Nat Commun. 2024 Feb 21;15:1596. doi: 10.1038/s41467-024-45497-4 (PMC10882007; doi:10.1038/s41467-024-45497-4)
Supplement: Supplementary file 3 — Description of Additional Supplementary Files [file 41467_2024_45497_MOESM3_ESM.pdf]

Description of Additional Supplementary Files

File Name: Supplementary Movie 1
Description: RTP phenomena and mechanical properties of the 3D printed lattice structures at different photocuring times.

File Name: Supplementary Movie 2
Description: RTP phenomena and mechanical properties of the 3D printed lattice structures after different humid treatments.

File Name: Supplementary Movie 3
Description: Failure monitoring of local mechanical properties of 3D printed table structure.
